# New insights into posttranslational modifications of proteins during bull sperm capacitation

**DOI:** 10.1186/s12964-023-01080-w

**Published:** 2023-04-12

**Authors:** Agnieszka Mostek-Majewska, Anna Majewska, Anna Janta, Andrzej Ciereszko

**Affiliations:** Department of Gamete and Embryo Biology, Institute of Animal Reproduction and Food Research of Polish Academy of Sciences, 10-748 Olsztyn, Poland

**Keywords:** Sperm, Capacitation, Bull, S-nitrosylation, S-glutathionylation, Tyrosine phosphorylation, Peroxyredoxin

## Abstract

**Background:**

Due to the unique nature of spermatozoa, which are transcriptionally and translationally silent, the regulation of capacitation is based on the formation of posttranslational modifications of proteins (PTMs). However, the interactions between different types of PTMs during the capacitation remain unclear. Therefore, we aimed to unravel the PTM-based regulation of sperm capacitation by considering the relationship between tyrosine phosphorylation and reversible oxidative PTMs (oxPTMs), i.e., S-nitrosylation and S-glutathionylation. Since reversible oxPTMs may be closely related to peroxyredoxin (PRDX) activity, the second aim was to verify the role of PRDXs in the PTM-based regulation of capacitation.

**Methods:**

Cryopreserved bull sperm were capacitated in vitro with or without PRDX inhibitor. Qualitative parameters of sperm and symptoms characteristic of capacitation were analyzed. Posttranslational protein modifications (S-nitrosylation, S-glutathionylation, tyrosine phosphorylation) were investigated at the cellular level (flow cytometry, fluorescence microscopy) and at the proteomic level (fluorescent gel-based proteomic approach).

**Results:**

Zona-pellucida binding proteins (ACRBP, SPAM1, ZAN, ZPBP1 and IZUMO4) were particularly rich in reversible oxPTMs. Moreover, numerous flagellar proteins were associated with all analyzed types of PTMs, which indicates that the direction of posttranslational modifications was integrated. Inhibition of PRDX activity during capacitation caused an increase in S-nitrosylation and S-glutathionylation and a decrease in tyrosine phosphorylation. Inhibition of PRDXs caused GAPDHS to undergo S-glutathionylation and the GSTO2 and SOD2 enzymes to undergo denitrosylation. Moreover, PRDX inhibition caused the AKAP proteins to be dephosphorylated.

**Conclusions:**

Our research provides evidence that crosstalk occurs between tyrosine phosphorylation and reversible oxPTMs during bull sperm capacitation. This study demonstrates that capacitation triggers S-nitrosylation and S-glutathionylation (and reverse reactions) of zona-pellucida binding proteins, which may be a new important mechanism that determines the interaction between sperms and oocytes. Moreover, TCA-related and flagellar proteins, which are particularly rich in PTMs, may play a key role in sperm capacitation. We propose that the deglutathionylation of ODFs and IZUMO4 proteins is a new hallmark of bull sperm capacitation. The obtained results indicate a relationship between PRDX activity and protein phosphorylation, S-glutathionylation and S-nitrosylation. The activity of PRDXs may be crucial for maintaining redox balance and for providing proper PKA-mediated protein phosphorylation during capacitation.

**Video Abstract**

**Supplementary Information:**

The online version contains supplementary material available at 10.1186/s12964-023-01080-w.

## Background

The sperm that are released from the testes are functionally immature. To become fully functional and perform fertilization, sperm must undergo capacitation, a post-testicular maturation process in the female reproductive tract. Since sperm cells are transcriptionally and translationally silent, this acquisition of sperm function is highly dependent on posttranslational modifications of proteins (PTMs) [1].

Among the known PTMs, tyrosine phosphorylation events are recognized as the most important hallmark of sperm capacitation. Proteomic studies revealed the molecular mechanisms that underly tyrosine phosphorylation and accompany capacitation in humans [2], mice [3], boars [4] and bulls [5]. Among different sperm structures, the flagellum may be the major component of tyrosine phosphorylation due to capacitation, which is strongly related to the binding capacity of sperm–zona pellucida [6]. Moreover, it was shown that sperm flagellar proteins undergo tyrosine phosphorylation and that this process is related to the acquisition of hyperactive motility [7]. In human spermatozoa, the protein A-kinase anchoring proteins (AKAPs) localized on the fibrous sheath are the most prominent tyrosine phosphorylated proteins during capacitation [8].

In contrast to phosphorylation, S-glutathionylation and S-nitrosylation have not been thoroughly investigated for their role in sperm physiology. However, there are strong indications that these reversible oxidative posttranslational modifications (oxPTMs) play an important role in the capacitation process [9, 10]. S-glutathionylation and S-nitrosylation are ubiquitous reversible modifications of oxPTMs and are recognized as the main processes in redox signaling in somatic cells; in addition, these modified oxPTMs were suggested to be the most likely oxPTMs capable of transmitting the signal in sperm capacitation [10]. It was shown previously that S-glutathionylation and S-nitrosylation regulate protein activity, stability, localization, and protein–protein interactions across myriad physiological processes, and their redox signaling potential is compared to that of phosphorylation/phosphorylation processes [11, 12].

Although it has been widely shown that sperm capacitation is accompanied by protein tyrosine phosphorylation, the links between tyrosine phosphorylation and other PTMs remain unknown. Our recent studies indicated that capacitation is associated with reversible oxPTMs [10]; however, which type of oxPTMs plays the dominant role in capacitation remains unclear. Moreover, our previous studies showed that peroxiredoxin (PRDX) activity is crucial to sperm motility and fertilizing ability, but its relationship to capacitation remains unclear [10]. Therefore, the aim of this study was to clarify the regulation of capacitation, including the relationship of tyrosine phosphorylation with protein S-nitrosylation and S-glutathionylation. The second aim was to verify the role of PRDXs in PTM-based regulation of capacitation. To verify the PTMs that accompany bull sperm capacitation and the role of PRDXs in this process, non-capacitated sperm (control), capacitated sperm and capacitated sperm with PRDXs inhibition were compared. Tyrosine phosphorylation, S-nitrosylation and S-glutathionylation were analyzed, both at the cellular level (flow cytometry and fluorescence microscopy analysis) and at the protein level (redox proteomics and Western blot analysis), to effectively visualize and find links between the studied modifications and sperm capacitation. In this study we were focused on characterization of frozen/thawed semen used in practical cattle breeding conditions.

## Methods

### Research material

Cryopreserved semen was obtained from sexually mature Holstein Friesian bulls (n = 8, in each experimental group) from the Mazowieckie Center for Animal Breeding and Reproduction (Łowicz, Poland). Unless otherwise declared, all the reagents were purchased from the Sigma‒Aldrich Chemical Company (St. Louis, MO, USA).

### Sperm preparation and capacitation

Prior to the analyses, cryopreserved semen straws were thawed for 60 s in a water bath brought to 38 °C. Thawed spermatozoa were separated from the diluent by centrifugation (360 × g for 10 min), and the sperm pellet was washed once with non-capacitating BO-SemenPrep medium (IVF Bioscience, Falmouth, UK). The pellets were then gently resuspended in the appropriate buffer to a concentration of 25 × 10^6^ cells/ml. To properly adjust the sperm concentration, the Count & Viability Kit (Luminex) in a GUAVA easyCyte 8HT Benchtop Flow Cytometer (Guava Technologies Inc., Luminex, Austin, TX, USA) was used as described below.

Sperm capacitation was carried out for 4 h at 38 °C in a 5% CO_2_ humidified atmosphere. The samples were suspended in capacitation buffer containing 100 mM NaCl, 3.1 mM KCl, 0.3 mM Na_2_HPO_4_, 21.6 mM Na-lactate, 0.4 mM MgCl_2_ × 4H_2_O, 10 mM HEPES, 1 mM Na-pyruvate, 50 µg/mL gentamycin and 6 mg/ml BSA (fraction V) with the addition of the capacitation inducers 2 mM CaCl_2_, 10 µg/mL heparin Fhyperand 25 mM NaHCO_3_ [13] in the presence (Cap PRDX−) or absence (Cap PRDX+) of 20 mM peroxiredoxin inhibitor Conoidin A (Merck, Darmstadt, Germany). The control samples (Non-Cap) were suspended in medium without capacitation inducers and with lower (5 mM) NaHCO_3_ content and were immediately collected by centrifugation. The progress of capacitation was evaluated after 4 h of incubation by examining the intracellular calcium level, actin polymerization progress and available acrosine level.

### Sperm motility measurement

Measurements were performed after thawing the sperm for 60 s at 37 °C. Semen (4 μL) was placed onto a Leja glass slide (IMV Technologies Group, Nieuw-Vennep, The Netherlands), which was then mounted on a heated stage (37 °C). The sperm motility was evaluated using a computer-assisted sperm analysis system HT CASA II (CEROS II system, Hamilton-Thorne, USA) as previously described [10]. A minimum of 200 events per replicate were recorded and measured using frame capture speed 60 Hz (30 frames). The following sperm characteristics were assessed: sperm motility (%), sperm progressive motility (%); amplitude of lateral head displacement (ALH); linearity of the movement (LIN); average path velocity (VAP); straight line velocity (VSL); and curvilinear velocity (VCL). At least 200 sperm cells per sample were measured.

### Flow cytometry and fluorescence microscopy analyses

Sperm aliquots from each experimental condition were labeled with different fluorescent probes and analyzed using a flow cytometer to evaluate sperm viability, nitric oxide content, intracellular calcium level, actin polymerization, acrosine availability, protein tyrosine phosphorylation, S-nitrosylation and S-glutathionylation. All cytometric analyses were performed with a GUAVA easyCyte 8HT Benchtop Flow Cytometer (Guava Technologies Inc., Luminex, Austin, TX, USA) with InCyte guavaSoft 4.0 software for data acquisition and analysis. Prior to the cytometric analysis, all fluorescently labeled samples were resuspended in PBS to obtain a final cell concentration of 350 cells/µl and placed in a 96-well plastic F-bottom clear plate (Greiner Bio-one, Frickenhausen, Germany). Forward scatter (FSC) and side scatter (SSC) signals were used to discriminate spermatozoa from debris, and 5 000 cells were acquired for each analysis. The localization of certain fluorescent probes in sperm cells was visualized using fluorescence microscopy with an Axio Observer. Z1/7 fluorescence microscope (Carl Zeiss, Inc., Oberkochen, Germany) equipped with ZEN 2.3 blue edition software (Carl Zeiss).

### Sperm count and viability

The sperm count and viability were measured using a Count & Viability Kit (Luminex), which utilizes a membrane-permeant DNA-staining dye based on 7-aminoactinomycin D. The fluorescent dye binds to the nuclei of the nonviable cells with increased membrane permeability. The additional dye included in the provided reagent stains only the nucleated cells, which allows us to exclude debris from the analysis. In brief, sperm aliquots were taken from the samples and diluted in fresh buffer to obtain 1 × 10^6^ cells/ml. Ten microliters of the suspension was mixed with 190 µl of the Count & Viability Reagent. After 5 min of incubation in the dark at room temperature, data were collected using a Guava cytometer. The fluorescence was excited by a blue 488 nm laser. The combination of two filters was applied to discriminate viable from nonviable cells (Yellow-B 583/26 nm) and nucleated cells from debris (Red-B 695/50 nm).

### Detection of intracellular nitric oxide levels

A Cell Meter™ Fluorimetric Intracellular Nitric Oxide (NO) Assay Kit (AAT Bioquest, Sunnyvale, CA, USA) was used to monitor intracellular nitric oxide levels in sperm cells according to the manufacturer’s protocol. Cell aliquots were taken from the sperm samples and incubated for 30 min at 38 °C in a 5% CO_2_ incubator with the provided Nitrixyte NIR fluorescent probe at a 1:500 dilution. Prior to the analysis, fluorescently labeled samples were resuspended in PBS to obtain a final cell concentration of 350 cells/µl. A red 642 nm laser was used to excite the fluorescence of the labeled cells, and the emitted light was captured using a Red-R 661/15 nm filter.

### Detection of intracellular calcium levels

The cell-permeant calcium indicator Fluo 3-AM (Sigma‒Aldrich), which exhibits an increase in fluorescence upon Ca^2+^ binding, was used to evaluate sperm intracellular calcium levels. A DMSO stock solution of Fluo3-AM fluorescent dye was added to the sperm aliquots to a final dye concentration of 2 µM and kept at 38 °C in a 5% CO_2_ incubator for 15 min. The cells were then diluted in PBS to obtain a final concentration of 350 cells/µl and immediately analyzed in a Guava cytometer. A blue 488 nm laser was used to excite the fluorescence of the labeled cells, and the emitted light was captured using a Green-B 525/30 nm filter.

### Actin polymerization

The fluorescent agent FITC-phalloidin, which stains F-actin filaments, was used to measure actin polymerization in sperm cells during capacitation. Sperm samples were collected by centrifugation (360 × g for 5 min), washed twice in PBS and fixed with 1% PFA in PBS (Santa Cruz Biotechnology Inc., Dallas, TX, USA) for 10 min. After fixation, the cell suspensions were washed twice again and then permeabilized with 0.1% Triton X-100 for 5 min. After another two washes, the cells were resuspended in PBS with 1% BSA to block nonspecific binding sites. After 20 min of incubation, the cells were centrifuged and resuspended in PBS supplemented with 1 µM FITC-labeled phalloidin reagent (Sigma‒Aldrich) and incubated in the dark for 10 min at room temperature. Prior to the analysis in the Guava cytometer, the sperm were diluted in PBS to a final concentration of 350 cells/µl. A blue 488 nm laser was used to excite the fluorescence of the labeled cells, and the emitted light was captured using a Green-B 525/30 nm filter.

### Phosphotyrosine and acrosine availability detection

To measure the protein phosphotyrosine and acrosine availability, cell aliquots were fixed, permeabilized and blocked as described above (section actin polymerization). Subsequently, the samples were incubated for 1 h with specific anti-phosphotyrosine (4G10 Platinum, Sigma‒Aldrich) or anti-acrosine (ACR-2, Exbio, Praha, Czech Republic) primary antibodies diluted 1:100 in PBS with 1% BSA. After two washes in PBS, sperm cells were incubated with FITC-conjugated anti-mouse IgG secondary antibodies (Sigma‒Aldrich) diluted 1:100 in PBS with 1% BSA for another hour. Before the analyses, sperm cells were washed twice in PBS and diluted to a final concentration of 350 cells/µl. A blue 488 nm laser was used to excite the fluorescence of the labeled cells, and the emitted light was captured using a Green-B 525/30 nm filter.

### Detection of S-glutathionylation at the cellular level

The level of protein S-glutathionylation in sperm cells was measured using an S-Glutathionylated Protein Detection Kit (Cayman Chemical, Ann Arbor, MI, USA) according to the manufacturer’s protocol. In brief, cell aliquots fixed with 1% PFA in PBS as described above (section actin polymerization) were washed twice in PBS and resuspended in PSSG Blocking Agent for 30 min to block free thiols in each sample. Afterward, the cells were incubated with PSSG Reduction Reagent for 15 min at 37 °C, then for 1 h in PSSG Labeling Reagent and finally for 1 h with PSSG Detection Reagent II (FITC). Each incubation step was followed by extensive washing of the cells with the assay buffer provided in the kit. Prior to the cytometric analysis, the samples were diluted to a final concentration of 350 cells/µl. A blue 488 nm laser was used to excite the fluorescence of the labeled cells, and the emitted light was captured using a Green-B 525/30 nm filter.

### Protein extraction and concentration measurement

The sperm cells from each sample were centrifuged at 900 × g/10 min/4 °C and washed with phosphate-buffered saline (PBS). After the supernatant was discarded, the samples were resuspended in lysis buffer (7 M urea, 2 M thiourea, 4% (w/v) 3-((3-cholamidopropyl)-dimethylammonio)-1-propanesulfonate (CHAPS), 2.5% (v/v) protease inhibitor cocktail, 1% (v/v) phosphate inhibitor cocktail, and 0.1 mM neocuproine) and subsequently sonicated on ice four times for 5 s at 30% amplitude using a VCX-130 Ultrasonic Processor (Sonics & Materials, Inc., Newtown, CT, USA). Prior to protein concentration measurement, samples were centrifuged at 14,000 × g for 5 min at 4 °C. Next, the supernatant was discarded, and pellets were resuspended in rehydration buffer (7 M urea, 2 M thiourea, 4% CHAPS). The protein concentration was measured using the Pierce 660 nm Protein Assay according to the manufacturer’s instructions (Thermo Scientific).

### Detection of S-nitrosylated proteins

To block all free thiols, 50 µg of each protein sample was mixed together in a 1:1 ratio with 50 mM N-ethylmaleimide (NEM) blocking buffer (100 mM Tris–HCl, pH 6.8, 1% SDS, 0.1 mM neocuproine) and incubated for 30 min at 37 °C. Excess NEM was removed using Zeba Spin Desalting Columns, 7 K MWCO (Thermo Scientific), according to the manufacturer’s protocol. To reduce S-nitrosylated proteins, the samples were incubated in a 1:1 ratio with substrate-specific reducing buffer (1 mM ascorbate in rehydratation buffer) for one hour at room temperature. After excess reducing buffer was removed with Zeba Spin Desalting Columns, the samples were prepared for the labeling step of all the endogenously available S-nitrosylated cysteine residues. Labeling of S-nitrosylated proteins was performed with the reagents included in the Saturn-2D REDOX Labeling Kit (DyeAGNOSTICS, Halle, Germany). Briefly, 5 μg of each protein sample was mixed with 5 μL of redox labeling buffer and labeled with 5 µl of S-Dye300. After one hour of incubation at 35 °C, the labeling reaction was stopped with 14 μL redox stop solution for 10 min. Prior to gel electrophoresis, each sample was mixed with the same quantity of the internal standard of proteins and run simultaneously on a single gel. An internal protein standard was prepared analogously by labeling the pool of all samples from the experiment with a maleimide-based dye (S-Dye200), which served as a further reference to allow gel–gel normalization. All steps were performed in the dark or protected from light.

### Detection of S-glutathionylated proteins

Free thiols were initially blocked using 50 mM NEM blocking buffer (100 mM Tris–HCl, pH 6.8, 1% SDS, 0.1 mM neocuproine). Fifty micrograms of each protein sample was mixed in a 1:1 ratio with NEM blocking buffer and incubated for 30 min at 37 °C. Then, excess NEM was removed using Zeba Spin Desalting Columns, according to the manufacturer’s protocol. Next, to reduce S-glutathionylated cysteine groups, samples were incubated for 30 min at room temperature in a 1:1 ratio with substrate-specific reducing buffer (13.5 μg/ml recombinant human glutaredoxin 1 (Grx1), 35 μg/ml oxidized glutathione (GSSG) reductase, 1 mM glutathione (GSH), 1 mM nicotinamide adenine dinucleotide phosphate (NADPH), 18 μM ethylenediaminetetraacetic acid disodium salt solution (EDTA), and 137 mM Tris–HCl, pH 8.0). Subsequently, excess reducing buffer was removed with Zeba Spin Desalting Columns, and samples were prepared for the labeling of all endogenously available S-glutathionylated cysteine residues. The labeling procedure was performed as described for S-nitrosylated proteins.

### Negative and positive controls of the labeling procedure

To determine the success of the blocking step, negative control samples were used in the absence of substrate-specific reducer in reducing buffer. In the case of protein S-nitrosylation, the control sample was performed in the absence of ascorbate, whereas in the case of S-glutathionylation, recombinant human glutaredoxin 1 was omitted. As a positive control, samples were additionally treated with 1 mM NONOate, a nitric oxide donor, and 5 mM GSSG, an oxidized glutathione donor, prior to the blocking step with NEM (Additional file 1: Fig. S1, Additional file 2: Fig. S2). The labeling procedure was performed as described above. Next, 5 μL of 2 × Laemmli loading buffer containing 80 mM DTT as a reductant was added. Next, 7 μL of each pooled sample containing 1 μg of protein was loaded in each lane. The proteins were resolved in 12.5% polyacrylamide gels during standard SDS‒PAGE electrophoresis and scanned.

### 2D-PAGE separation of sperm proteins and protein identification

Five micrograms of each protein sample was selected for 2D-PAGE separation and mixed with 5 μg of the internal standard proteins, then rehydration buffer was added to a volume of 340 μL. The samples were loaded onto 18 cm Immobiline DryStrips with a nonlinear 3–10 pH gradient (GE Healthcare, Chicago, IL, USA) and rehydrated for 12 h. Isoelectric focusing was performed as previously described [10]. Moreover, the strips were equilibrated in SDS equilibration buffer (6 M urea, 75 mM Tris–HCl (pH 8.8), 29.3% glycerol, 2% SDS, and a trace amount of bromophenol blue) containing 10 mg/mL DTT for 15 min and subsequently in SDS equilibration buffer containing 25 mg/mL iodoacetamide for 15 min. The second dimension of 2D-PAGE was performed for 16 h at 1 W/gel utilizing an Ettan Dalt-Six apparatus (GE Healthcare).

Differential protein spots were manually excised and subjected to trypsin digestion and spectrometric identification with a MALDI Autoflex Speed TOF/TOF mass spectrometer (Bruker Daltonics) as previously described [10].

### Detection of tyrosine phosphorylated proteins

After 2D PAGE electrophoresis the gels were placed in a ChemiDoc Touch Imaging System (Bio-Rad) and activated for 45 s in order to enable stain-free visualization of the total proteins. The proteins were then electrotransferred from the gels onto a 0.45 µm nitrocellulose membranes (60 V for 2 h in a Bio-Rad Mini Protean II trans-blot cell operating in a cold room) and the stain-free quantitative images of the transferred proteins were captured in the ChemiDoc Touch Imaging System (Bio-Rad). Membranes were then blocked for 1 h in a 2% skim milk dissolved in a tris-buffered saline supplemented with 0.05% of Tween-20 (TBS-T), and incubated for 2 h in 1:2 000 anti-phosphotyrosine primary antibody (4G10 Platinum, Millipore). After extensive washing in TBS-T, the membranes were incubated for 1 h in 1:30 000 anti-mouse HRP Conjugate secondary antibodies (Bio-Rad). Following the extensive washing in TBS-T the membranes were developed with the Clarity Western ECL Substrate (Bio-Rad) and the chemiluminescence of the phosphotyrosines was captured in ChemiDoc Touch Imaging System (Bio-Rad). The obtained images were analyzed in the Image Lab 5.0 software (Bio-Rad) and the stain-free images of the total proteins transferred to the membranes were used to normalize the signal from the separate samples.

### Statistical analyses

The results are presented as the mean ± standard deviation (SD) (n = 8). All analyses were performed at a significance level of *p* < 0.05 using GraphPad Prism software v6.02 (GraphPad Software Inc. San Diego, CA, USA). Data expressed as a percentage were normalized by arcsine square root transformation. Data were analyzed using two-way repeated-measures ANOVA, followed by the Friedman test for post hoc comparison of means. To analyze protein gels, the statistical component of the SameSpots software was used, as described above.

## Results

### Sperm viability

**Fig. 1:**
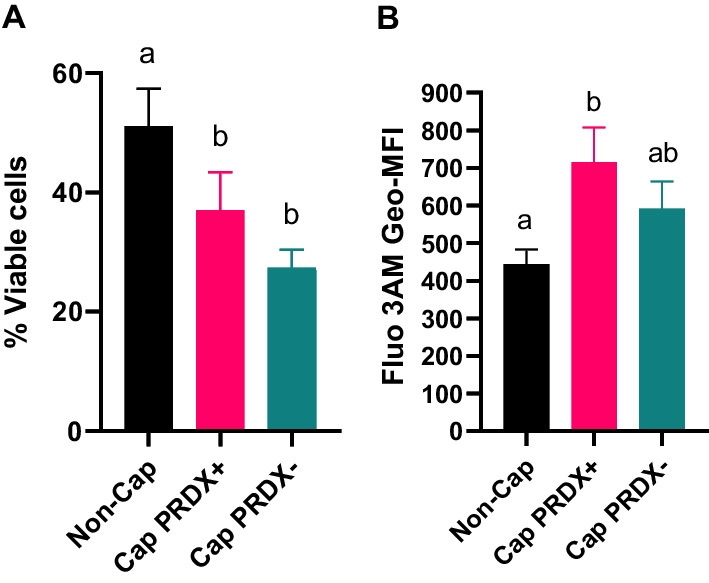
Percentage of live sperm (**a**) and intracellular calcium level (**b**) in non-capacitated sperm (Non-Cap), capacitated sperm (Cap PRDX+) and capacitated sperm with PRDX inhibition (Cap PRDX−). Viability and intracellular levels were measured by flow cytometry. Data are expressed as the mean ± SD, *p* < 0.05, (n = 8 in each group)

The level of sperm viability decreased as a result of capacitation by 24.0% and as a result of capacitation with inhibition of PRDX by 40% (Fig. 1a).

### Capacitation

The following parameters, which indicated the occurrence of capacitation in sperm, were analyzed: intracellular accumulation of calcium ions, increased acrosin availability and actin polymerization. Intracellular calcium levels were 55.5% higher in capacitated sperm than in non-capacitated sperm (*p* < 0.05) (Fig. 1b). The level of intracellular calcium in the capacitated sperm with PRDX inhibition did not differ significantly.

**Fig. 2:**
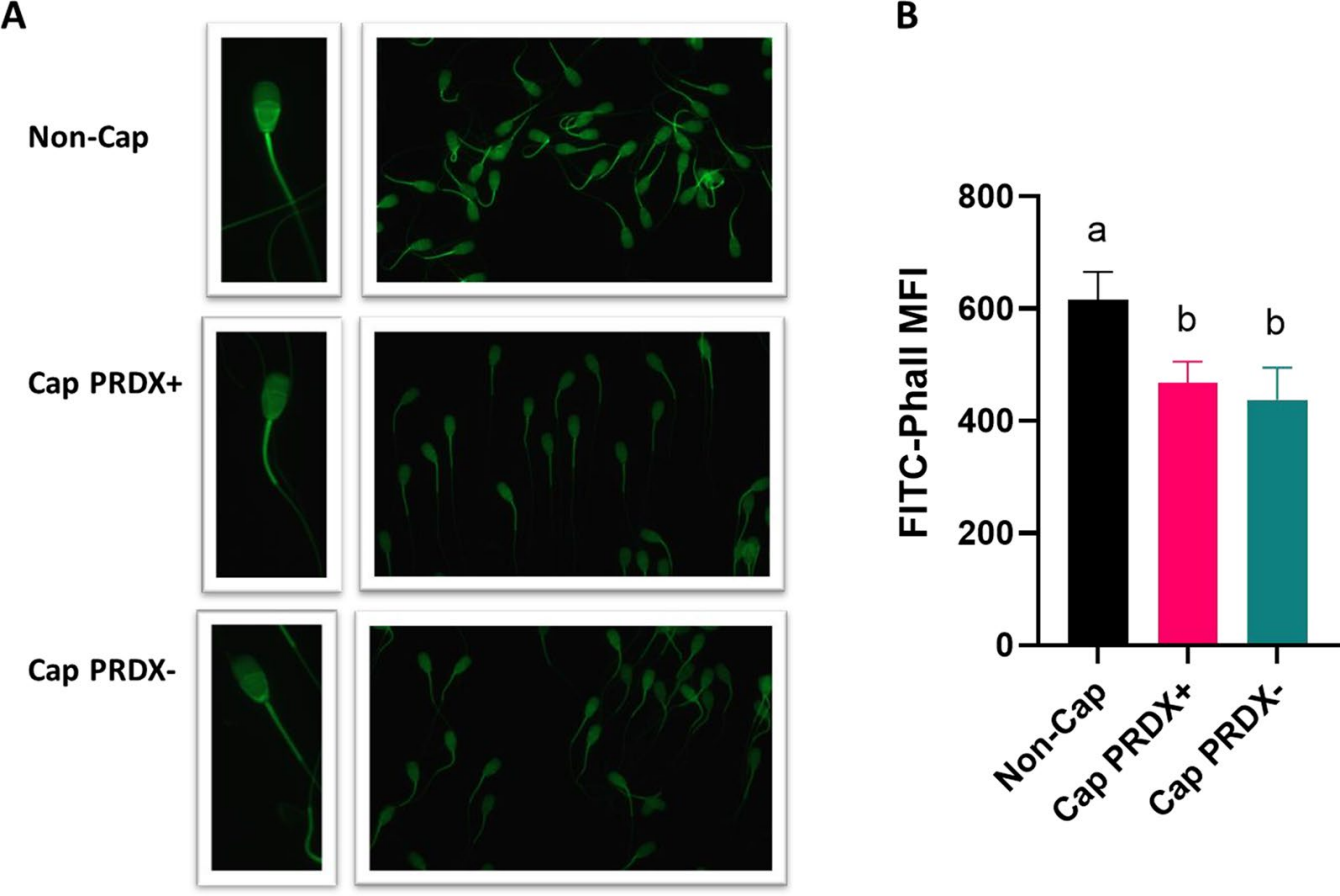
Representative fluorescence microscopy images (**a**) and flow cytometry graph (**b**) showing the location and level of F-actin in non-capacitated sperm (Non-Cap), capacitated sperm (Cap PRDX+) and capacitated sperm with PRDX inhibition (Cap PRDX−). Data are expressed as the mean ± SD, *p* < 0.05, (n = 8 in each group)

The most intense fluorescence signal of F-actin originated from the post acrosomal sperm region and the midpiece (Fig. 2a). Flow cytometry analysis showed that the level of F-actin decreased by 29.5% and 32.6% in capacitated sperm and capacitated sperm with PRDX inhibition, respectively (Fig. 2b).

**Fig. 3:**
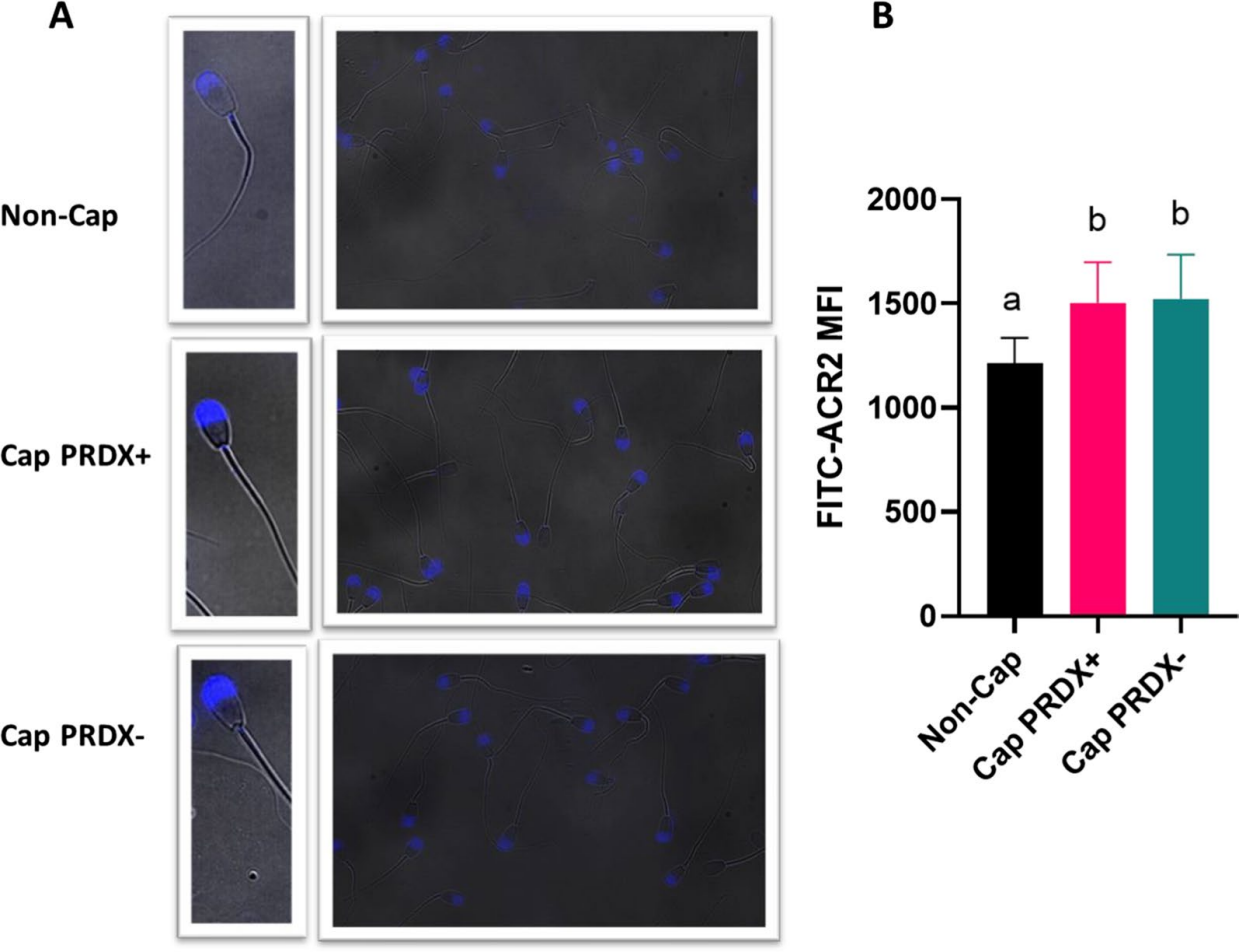
Representative fluorescence microscopy images (**a**) and flow cytometry graph (**b**) showing the location and level of available acrosine in non-capacitated sperm (Non-Cap), capacitated sperm (Cap PRDX+) and capacitated sperm with PRDX inhibition (Cap PRDX−). The data are expressed as the mean ± SD, *p* < 0.05, (n = 8 in each group)

Fluorescence microscopy images showed the presence of acrosine in the acrosomal sperm region in all observed sperm samples (Fig. 3a). Flow cytometry analysis showed that the level of available acrosine increased by 25.1% and 26.6% in capacitated sperm and capacitated sperm with PRDX inhibition, respectively (Fig. 3b).

### Sperm motility

Total and progressive sperm motility decreased in capacitated sperm and in capacitated sperm with PRDX inhibition by 25.9% and 85.6%, respectively, compared to that of non-capacitated sperm (*p* < 0.05). Movement trajectory parameters, including ALH, VAP, VCL and VSL, were decreased only in capacitated sperm with PRDX inhibition. In turn, BCF, LIN and STR remained unchanged (Table 1).

**Table 1:** Bull sperm motility parameters of non-capacitated sperm (Non-Cap), capacitated sperm (Cap PRDX+) and capacitated sperm with PRDX inhibition (Cap PRDX−)

	Non-Cap	Cap PRDX+	Cap PRDX−
Total motility (%)	50.1 ± 5.8^a^	37.1 ± 7.8^b^	7.2 ± 2.7^c^
Progressive motility (%)	41.5 ± 6.6^a^	29.8 ± 8.1^b^	4.0 ± 1.8^c^
ALH (μm)	8.0 ± 0.5^a^	7.5 ± 0.6^a^	5.1 ± 1.1^b^
BCF (Hz)	26.0 ± 3.3^a^	28.5 ± 2.5^a^	32.9 ± 5.0^a^
LIN (%)	60.4 ± 4.7^a^	63.6 ± 3.9^a^	57.7 ± 8.8^a^
STR (%)	88.9 ± 3.2^a^	89.8 ± 3.2^a^	85.5 ± 5.3^a^
VAP (μm s^−1^)	134.8 ± 12.0^a^	139.4 ± 7.0^a^	80.6 ± 7.1^b^
VCL (μm s^−1^)	201.3 ± 7.8^a^	198.0 ± 6.7^a^	123.0 ± 28.4^b^
VSL (μm s^−1^)	121.5 ± 14.6^a^	127.7 ± 9.6^a^	69.9 ± 15.7^b^

The values are presented as the mean ± SD. A lack of superscript letters indicates no significant differences (*p* < 0.05) in sperm quality parameters between treatments

*MOT* Motility, *ALH* Amplitude of lateral head displacement, *BCF* Beat cross frequency, *LIN* Linearity, *STR* Straight-line velocity, *VAP* Average path velocity, *VCL* Curvilinear velocity, *VSL* Straight line velocity

### Detection of nitric oxide in sperm

**Fig. 4:**
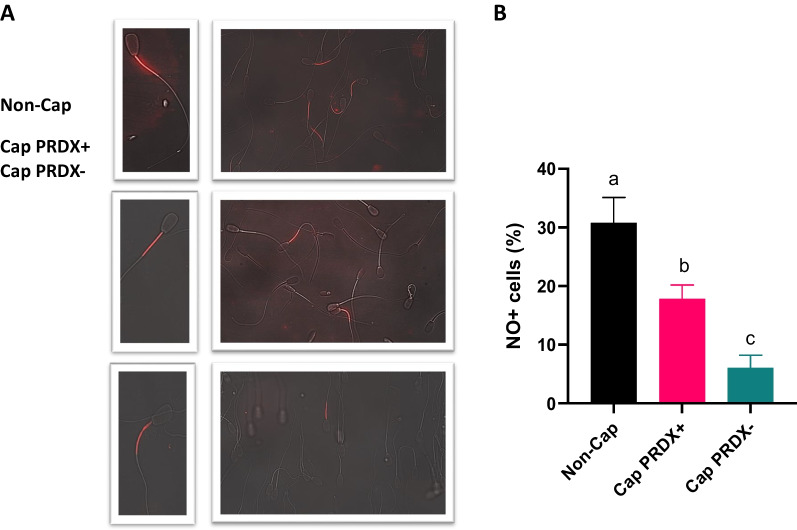
Representative fluorescence microscopy images (**a**) and flow cytometry graph (**b**) showing the location and level of intracellular nitric oxide in non-capacitated sperm (Non-Cap), capacitated sperm (Cap PRDX+) and capacitated sperm with PRDX inhibition (Cap PRDX−). The data are expressed as the mean ± SD, *p* < 0.05, (n = 8 in each group)

Fluorescence microscopy images showed that intracellular NO in bull sperm accumulated in the sperm midpiece, indicating a mitochondrial origin (Fig. 4a). Cytometric analysis showed that the level of intracellular NO decreased by 39.3% in capacitated sperm and by 83.1% in capacitated sperm with PRDX inhibition, indicating that intracellular NO was used up in the cellular processes (Fig. 4b).

### Detection of S-glutathionylated proteins in sperm

**Fig. 5:**
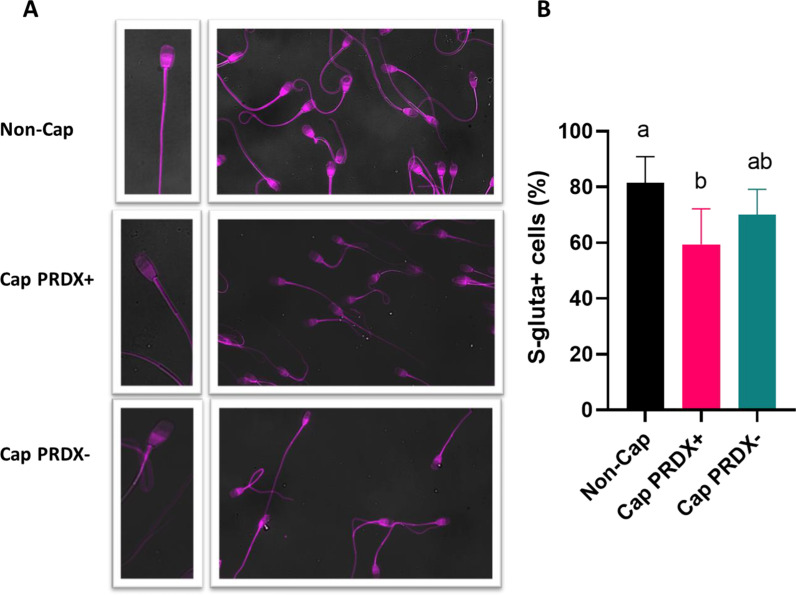
Representative fluorescence microscopy images (**a**) and flow cytometry graph (**b**) showing the location and level of protein S-glutathionylation in non-capacitated sperm (Non-Cap), capacitated sperm (Cap PRDX+) and capacitated sperm with PRDX inhibition (Cap PRDX−). Data are expressed as the mean ± SD, *p* < 0.05, (n = 8 in each group)

Fluorescence microscopy images showed the presence of S-glutathionylated proteins in all parts of the sperm (sperm flagellum and sperm head at both, postequatorial and postacrosomal regions). The most intense fluorescence signal comes from the postequatorial and postacrosomal sperm regions (Fig. 5a). Flow cytometry analysis showed that the level of protein S-glutathionylation decreased by 27.1% in capacitated sperm. The level of S-glutathionylation in the capacitated sperm with PRDX inhibition did not differ significantly (Fig. 5b).

### Detection of tyrosine phosphorylation in sperm

**Fig. 6:**
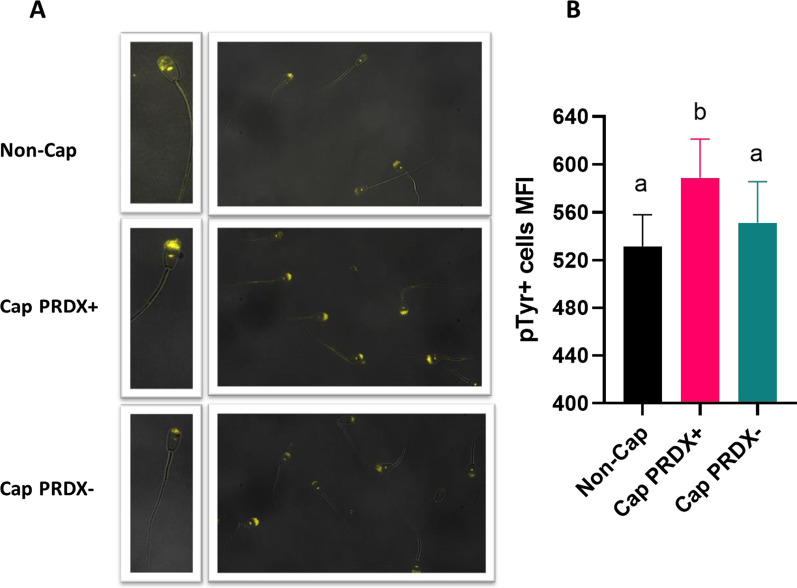
Representative fluorescence microscopy images (**a**) and flow cytometry graph (**b**) showing the location and level of tyrosine phosphorylation in non-capacitated sperm (Non-Cap), capacitated sperm (Cap PRDX+) and capacitated sperm with PRDX inhibition (Cap PRDX−). The data are expressed as the mean ± SD, *p* < 0.05, (n = 8 in each group)

The fluorescence signal indicating the presence of tyrosine-phosphorylated sperm was located variously in non-capacitated sperm, capacitated sperm and capacitated sperm with PRDX inhibition. In capacitated sperm, an intensely bright fluorescence signal was observed in the acrosomal region, in contrast to non-capacitated sperm and capacitated sperm with PRDX inhibition. However, in all sperm samples, a small bright spot was observed in the center of the equatorial segment (Fig. 6a). Flow cytometry analysis showed that the level of phosphorylation increased by 10.9% in capacitated sperm. The level of phosphorylation in the capacitated sperm with PRDX inhibition was lower than that in the capacitated sperm with active PRDXs and did not differ significantly from that in non-capacitated sperm (Fig. 6b).

### S-glutathionylation, S-nitrosylation and tyrosine phosphorylation occurred due to capacitation at the proteomic level

**Fig. 7:**
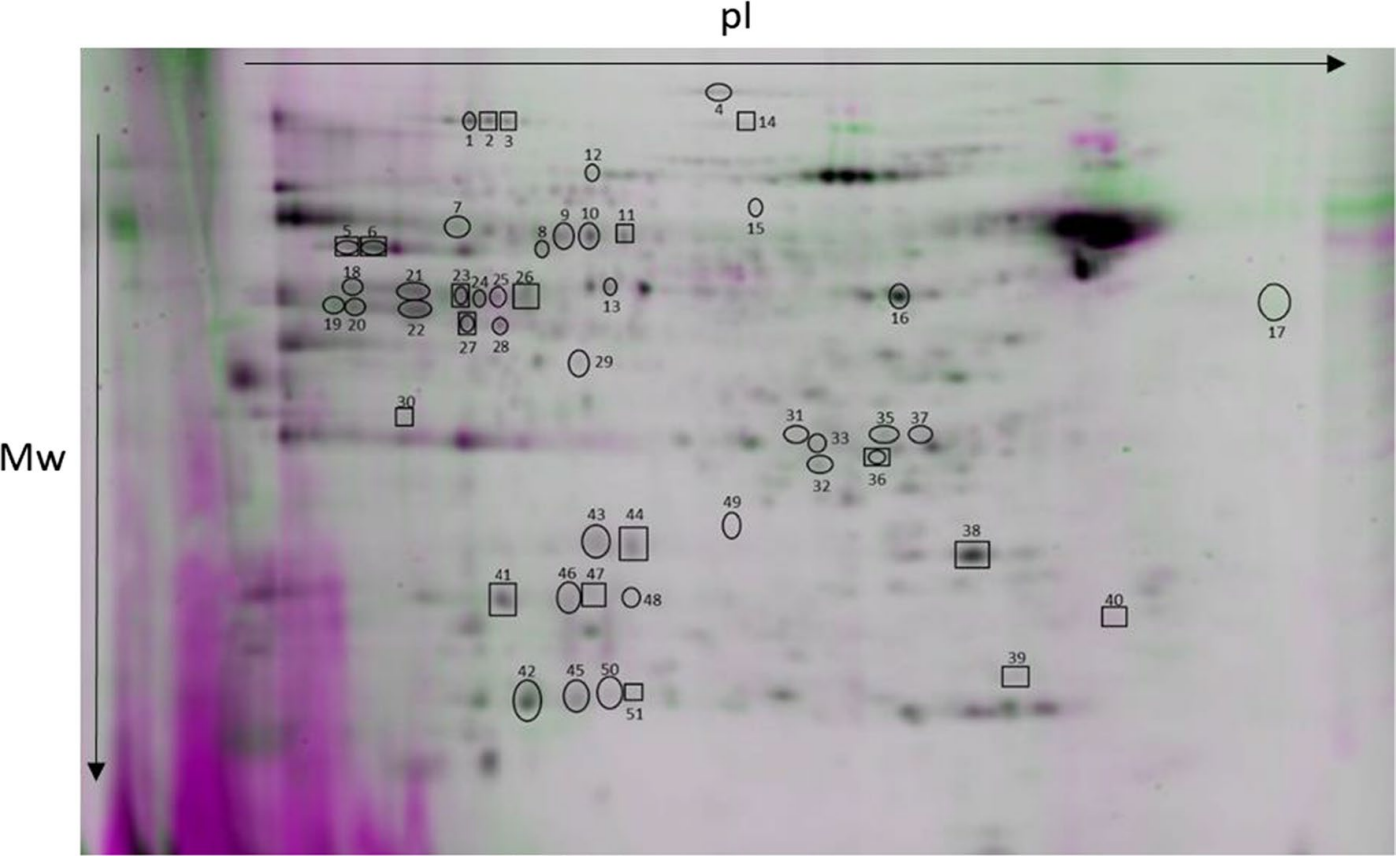
Representative 2D gel showing protein spots with changed levels of S-nitrosylation. Protein spots in which the level of S-nitrosylation was changed under the influence of capacitation are marked by circles. Protein spots in which the level of S-nitrosylation was changed due to PRDX inhibition are marked by rectangles. The direction of changes in the level of S-nitrosylation between treatments and protein names correspond to the data in Table 2

**Fig. 8:**
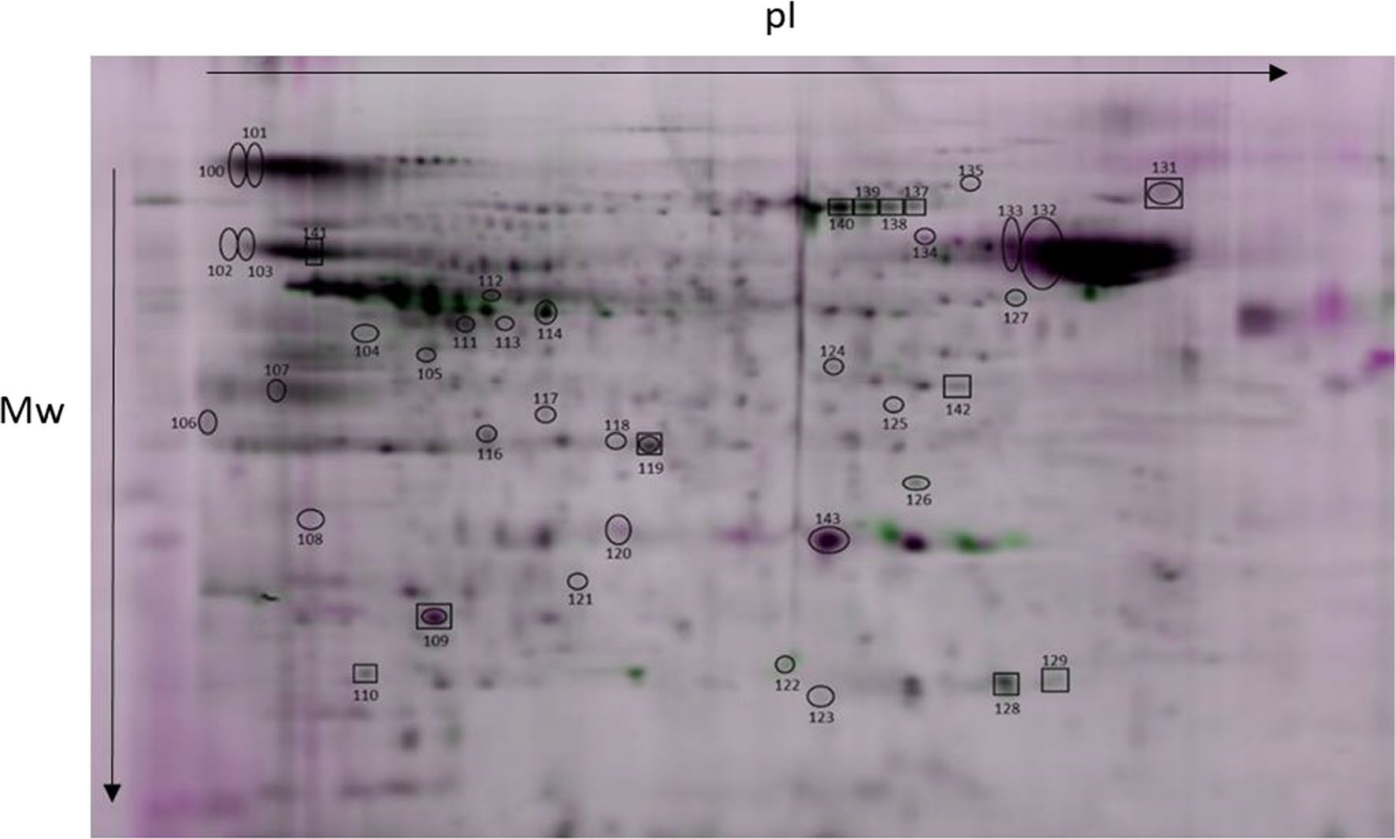
Representative 2D gel showing protein spots with changed levels of S-glutathionylation. Protein spots in which the level of S-glutathionylation was changed under the influence of capacitation are marked by circles. Protein spots in which the level of S-glutathionylation was changed due to PRDX inhibition are marked by rectangles. The direction of changes in the level of S-glutathionylation between treatments and protein names correspond to the data in Table 2

**Fig. 9:**
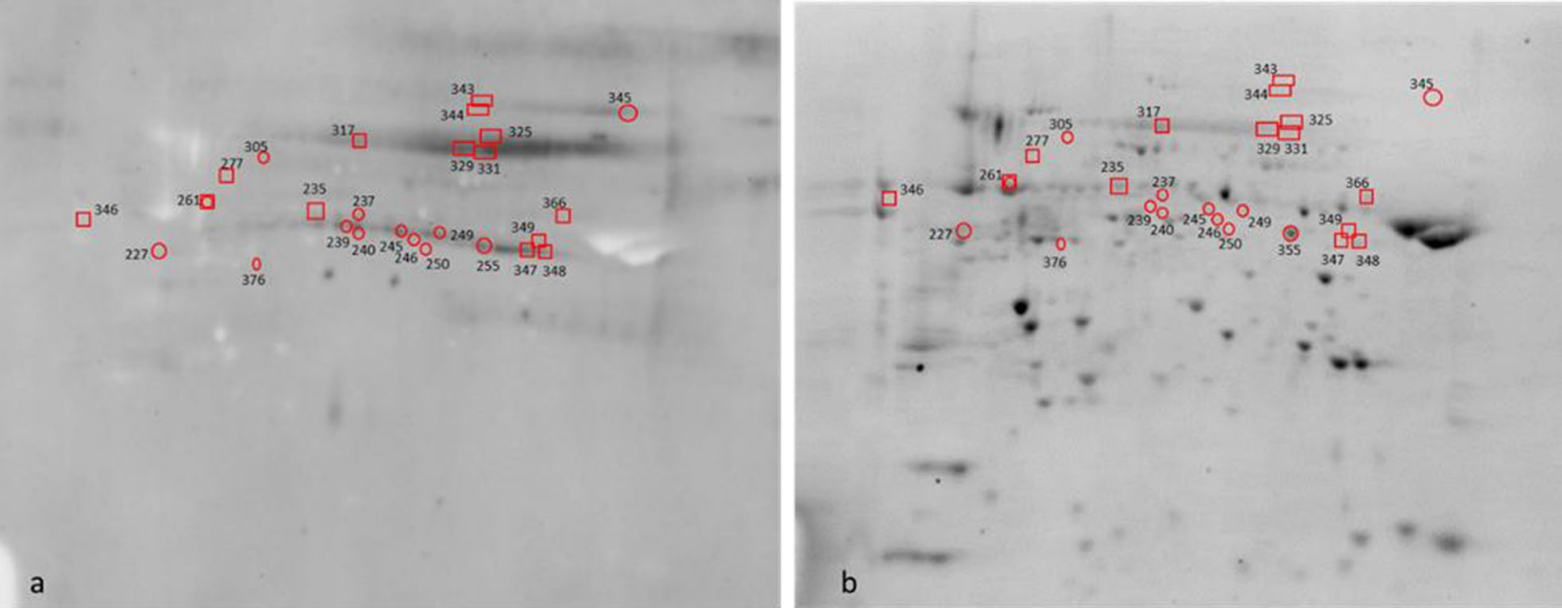
Representative Western blot (**a**) with corresponding stain-free gel (**b**) showing proteins with changed levels of tyrosine phosphorylation. Protein spots in which the level of phosphorylation was changed under the influence of capacitation are marked by circles. Protein spots in which the level of phosphorylation was changed due to PRDX inhibition are marked by rectangles. The direction of changes in the level of phosphorylation between treatments and protein names correspond to the data in Table 2

Fifty-two protein spots (corresponding to 28 proteins) that showed altered levels of S-nitrosylation were identified (Fig. 7, Table 2, Additional file 3: Table S1). Subsequently, 42 protein spots (corresponding to 26 proteins) in which the levels of S-glutathionylation was changed were identified (Fig. 8, Table 2, Additional file 3: Table S1). Moreover, 28 protein spots (corresponding to 21 proteins) that showed altered levels of tyrosine phosphorylation were identified (Fig. 9 and Table 2, Additional file 3: Table S1). Proteomic results showed that reversible PTMs occurred during capacitation with the following modifications: S-nitrosylation/denitrosylation, S-glutathionylation/deglutathionylation and tyrosine phosphorylation/dephosphorylation. The PTMs resulting from capacitation vary depending on whether the PRDXs are active or inhibited.

**Table 2:** Identified bull sperm proteins showing S-nitrosylation (S-nitro), denitrosylation (Denitro), S-glutathionylation (S-gluta), deglutathionylation (De-gluta), tyrosine phosphorylation (pTyr) and dephosphorylation (De-pTyr)

Name	Gene	S-nitro	Denitro	S-gluta	De-gluta	pTyr	De-pTyr
**TCA cycle**							
Aconitase 2, mitochondrial precursor	ACO2		1* 2* 3*				
2-Oxoglutarate dehydrogenase, mitochondrial precursor	OGDH	4					
Dihydrolipoyl dehydrogenase, mitochondrial	DLD		10* 11*			245* 249*	
Fumarate hydratase, mitochondrial	FH		22* 23* 24*	111* 112* 113*			
Citrate synthase, mitochondrial precursor	CS		27* 28*			376*	
Isocitrate dehydrogenase [NAD] subunit alpha, mitochondrial precursor	IDH3A			118*			
L-lactate dehydrogenase C chain isoform X2	LDHC				116*		
**Energy metabolism**							
ATP synthase subunit alpha, mitochondrial isoform X1	ATP5F1A		5* 6* 8*				
E Chain E, ATP synthase D chain, mitochondrial	ATP5ME				123*		
F chain F, ATP synthase subunit beta, mitochondrial	ATP5F1B			127			
C chain C, bovine mitochondrial F1-ATPase	1COW						261*
A chain A, cytochrome B-c1 complex subunit 1, mitochondrial	UQCRC1		16*			255*	
N chain N, cytochrome b-c1 complex subunit 1, mitochondrial	UQCRC1	21*					
B chain B, cytochrome B-c1 complex subunit 2, mitochondrial	UQCRC2		19*	104			
NADH dehydrogenase [ubiquinone] iron-sulfur protein 8, mitochondrial isoform X3	NDUFS8	39*					
C chain C, NADH dehydrogenase [ubiquinone] iron-sulfur protein 3, mitochondrial	NDUFS3				143*		
Pyruvate kinase PKM isoform X1	PKM		7*				
Alpha enolase	ENO1			114*		240*	
Serine/threonine-protein phosphatase PP1-gamma catalytic subunit isoform X1	PPP1CC		35* 37*			242*	
**Organelle component assembly (general)**							
MICOS complex subunit MIC60 isoform X4	IMMT		14*				
Prohibitin isoform X1	PHB			126			
Ras-related protein Rab-2A isoform X3	RAB2A				122*		
Ras-related protein Rab-2A	RAB2A		51*				
Transitional endoplasmic reticulum ATPase	VCP			130			
**Organelle component assembly (actin polymeryzation)**							
Actin-like protein 7A	ACTL7A		13*				
F-actin-capping protein subunit beta isoform X3	CAPZB	31*					
A chain A, actin, cytoplasmic 1	ACTB			142			
Fascin-3 isoform X2	FSCN3					234*	
**Fertilization**							
Disintegrin and metalloproteinase domain-containing protein	ADAM2	17					
Zonadhesin	ZAN		25* 26*				
Zona pellucida-binding protein 1 isoform X1 (sperm inner acrosomal membrane protein IAM38)	ZPBP1			106*			
Sperm adhesion molecule 1 isoform X2	SPAM1			100* 101*			
Izumo sperm-egg fusion protein 4	IZUMO4	43 44 49*	47* 48*		120* 121*		
Acrosin-binding protein isoform X1	ACRBP	38* 46*					
Sperm equatorial segment protein 1 precursor	SPESP1					250*	
Postacrosomal sheath WW domain-binding protein (PAWP)	WBP2NL		32*				
**Redox process**							
Glycerol-3-phosphate dehydrogenase, mitochondrial isoform X1	GPD2	12*		107*			
Glyceraldehyde-3-phosphate dehydrogenase, testis-specific isoform X1	GAPDHS		18* 20*	102 103 141			
Glutathione S-transferase omega-2 isoform X1	GSTO2		41*				
Superoxide dismutase [Mn], mitochondrial precursor	SOD2		42* 45* 50*				
L-amino-acid oxidase isoform X2	IL4I1						346
**Sperm flagellum**							
Tektin 3	TEKT3		9*				
Tektin-2 isoform X1	TEKT2					246*	
Radial spoke head 14 homolog isoform X1	RSPH14	33*					
Dynein, axonemal, light intermediate chain 1-like	DNALI1				108*		
Tubulin alpha-3 chain isoform X3	TUBA3A				132*		
Outer dense fiber protein 2 isoform X20	ODF2				124*	305* 329* 331*	
Outer dense fiber protein 2 isoform X17	ODF2					325*	
Nucleoside diphosphate kinase homolog 5 isoform X1	NME5		52*				
Phosphatidylethanolamine-binding protein	PEBP1				110*		
**Sperm flagellum (cAMP/PKA pathway)**							
H chain H, cAMP-dependent protein kinase type I-alpha regulatory subunit (PKA)	PRKAR1A					347* 348* 349*	
A-kinase anchor protein 4	AKAP4					317	343
A-kinase anchor protein 3	AKAP3						344
**Chaperonin**							
T-complex protein 1 subunit alpha isoform X1	TCP1		15*				
DnaJ homolog subfamily B member 13	DNAJB13		30*				
60 kDa heat shock protein, mitochondrial	HSPD1				133*	366	
Heat shock-related 70 kDa protein 2	HSPA2				135*		
TPA: T-complex protein 1 subunit zeta-2	CCT6B					277	
**Other**							
Casein kinase II subunit alpha	CSNK2A1			105			
B chain B, seryl-trna synthetase	SARS2					238*	
Fructose-1,6-bisphosphatase, partial	FBP1				117*		
Leucine rich repeat containing 37A-like protein	LRRC37A						345*
Ubiquitin carboxyl-terminal hydrolase isozyme L1 isoform X1	UCHL1		40*				
Cytosolic 5′-nucleotidase 1B isoform X2	NT5C1B					237*	
Cytosolic 5′-nucleotidase 1B isoform X1	NT5C1B					235*	
Sperm-associated antigen 6	SPAG6					239*	

The numbers of differential protein spots between non-capacitated sperm (Non-Cap) and capacitated sperm (Cap PRDX+) are marked with an asterisk. The numbers of differential protein spots between capacitated sperm with active (Cap PRDX+) and inhibited PRDXs (Cap PRDX−) are underlined

### Changes in PTMs due to capacitation with active PRDXs

**Fig. 10:**
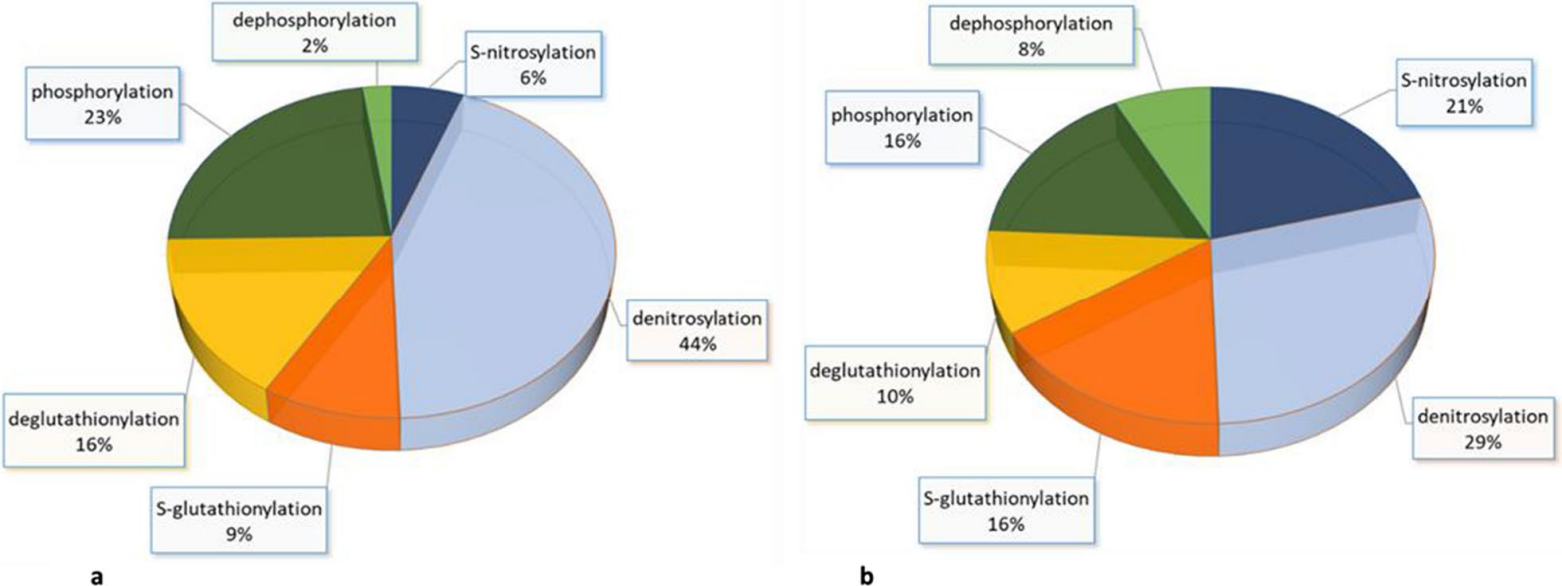
Pie diagram showing the contribution of selected PTMs of sperm proteins due to capacitation with active PRDXs (**a**) and inhibited PRDXs (**b**). A list of proteins that changed in terms of individual PTMs is provided in Table 2

The dominant PTMs detected in capacitated sperm proteins with active PRDXs were denitrosylation (reduction of S-nitrosylation in 44% of protein spots), deglutationylation (reduction in S-glutathionylation in 16% of protein spots) and tyrosine phosphorylation (increase in phosphorylation in 23 protein spots). In turn, S-nitrosylation, S-glutathionylation and dephosphorylation were the minorities (6%, 9% and 2% of protein spots, respectively) (Fig. 10a).

The most prominent group of proteins that underwent denitrosylation was associated with the TCA cycle and energy metabolism (18 protein spots). Deglutathionylation and tyrosine phosphorylation were most prominent modifications in the flagellar proteins (4 and 6 protein spots, respectively). The largest group of deglutathionylated and phosphorylated proteins was associated with sperm flagellum. The highest amount of phosphorylated proteoforms was detected in protein kinase A phosphorylation, which was higher in capacitated sperm (spots 347, 348, 349) (Table 2).

Zona pellucida binding proteins were characterized by the highest number of coexisting changes in the level of S-nitrosylation and S-glutathionylation in both directions. The highest number of S-nitrosylated proteoforms was detected in the IZUMO4 protein (5 proteoforms with changed S-nitrosylation levels between treatments corresponding to the following spots: 43, 44, 47, 48, 49) (Table 2).

### Changes in PTMs due to capacitation with inhibited PRDXs

Inhibition of PRDXs during sperm capacitation resulted in a 15% increase in S-nitrosylation, a 7% increase in S-glutathionylation, and a 7% decrease in protein phosphorylation compared to that of sperm with active PRDXs (Fig. 10b).

The increase in S-nitrosylation was most evident in proteins related to the TCA pathway (8 spots), fertilization (5 spots) and redox enzymes (4 spots). The increase in S-glutathionylation was most pronounced in proteins related to energy metabolism (5 spots) and redox enzymes (3 spots). In turn, a decrease in phosphorylation was detected in sperm-associated flagellum proteins (8 spots) (Table 2).

### Analysis of the interaction of S-nitrosylated, S-glutathionylated and phosphorylated proteins

**Fig. 11:**
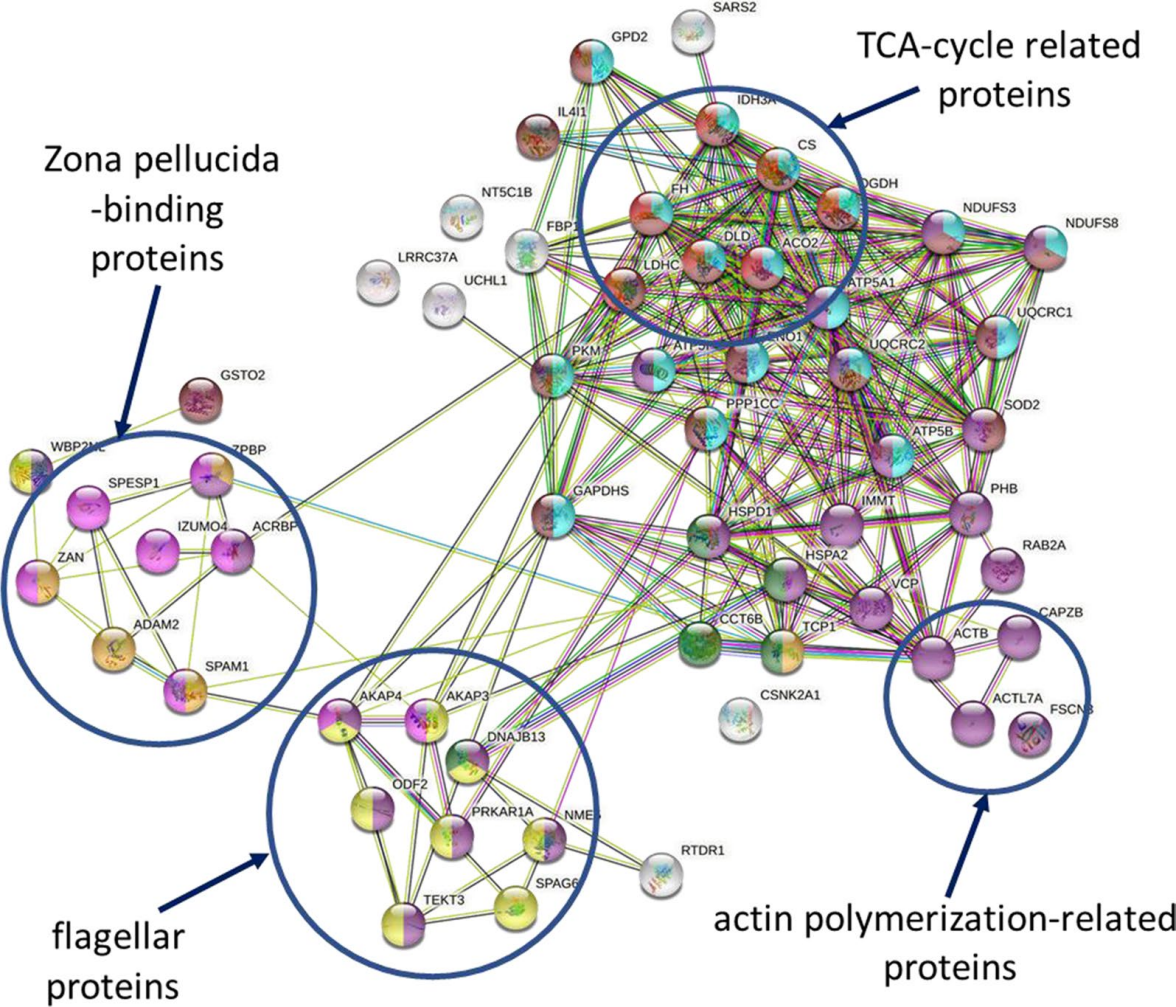
STRING protein association analysis of S-nitrosylated, S-glutathionylated and phosphorylated proteins listed in Table 2. Network nodes represent proteins, and the connecting lines represent protein‒protein interactions

STRING protein interaction analysis of identified proteins showed an associated groups of fertility-related proteins that undergo simultaneous S-nitrosylation, S-glutathionylation and phosphorylation. Proteins representing the largest associated group are responsible for binding sperm to zona pellucida (ADAM2, ZAN, ZPBP, SPAM1) sperm-egg fusion (IZUMO 4) and preparation for acrosome reaction (ACRBP, SPESP1, WBP2NL). The second group of related proteins is associated with actin polymerization (ACTL7A, CAPZB, ACTB, FSCN) (Fig. 11).

## Discussion

### A general examination of S-nitrosylation proteins, S-glutathionylation and tyrosine phosphorylation during sperm capacitation

Although it is known that mammalian sperm capacitation is a redox-regulated and phosphorylation-dependent process, little information is available on the biochemical connections between these PTMs. Previous research by our group demonstrated that the direction of reversible redox modifications (S-glutathionylation, S-nitrosylation, formation of disulfide bridges and sulfoxylation) during capacitation of fresh semen is mostly directed toward protein cysteine oxidation rather than reduction [10]. Interestingly, the current analysis of S-glutathionylation and S-nitrosylation as separate reversible oxPTMs showed that the overall level of these modifications at the cellular (Figs. 4, 5) and protein levels (Fig. 10a) was decreased due to capacitation compared to that of phosphorylation (Figs. 6, 10a). In other words, bull sperm capacitation was dominated by denitrosylation, deglutathionylation and tyrosine phosphorylation (Fig. 10), which indicates that the mechanism of capacitation regulation by PTMs is much more complex than previously assumed. It should be taken into account that the observed changes in sperm parameters and PTMs during capacitation of bull sperm refer to cryopreserved semen and may differ from changes in fresh semen due to cryogenic damage to the sperm. The results provide evidence that reduction and oxidation simultaneously occur within various reversible oxPTMs and that during capacitation, these processes can regulate the functional switches integral to homeostatic redox signal transduction. Such crosstalk between S-nitrosylation and S-glutathionylation was previously observed in somatic cells, especially in heart muscle [14]. However, the current results show for the first time that sperm capacitation is accompanied by a change in S-nitrosylation and S-glutathionylation (with a predominance of a reduction) levels and an increase in phosphorylation levels, providing an interesting convergence of cyclical posttranslational modifications.

In the following sections, the changes in PTMs that occur during the capacitation of bull sperm with active PRDXs will be discussed. The differences between PTM formation in capacitation with active and inhibited PRDXs are discussed in a separate section.

### Decrease of sperm viability during capacitation

Our results showed a decrease in viability and motility due to capacitation. When analyzing sperm viability during capacitation, it should be borne in mind that out of the entire population of spermatozoa, only a part undergoes capacitation [15]. Indeed, sperm that have survived the capacitation conditions are viable and have activated motility. The remaining spermatozoa undergo apoptosis over time and this is a natural phenomenon reflecting the situation of spermatozoa in the female reproductive tract. The timing of capacitation must also be taken into account—some spermatozoa are just beginning the capacitation process, while others—not being fertilized—undergo apoptosis. This phenomenon was explained by Aitken [16]. The reason for the decrease in motility are most likely reactive oxygen species produced by spermatozoa. ROS trigger a signaling cascade leading to capacitation in part of the sperm population, while in the sperm that are unable to cope with the increasing amount of ROS produced during capacitation undergo loss of motility and apoptosis [16].

### Capacitation-related PTMs of mitochondrial proteins

During the capacitation process, changes in the level of S-nitrosylation, S-glutathionylation and phosphorylation of associated groups of proteins occur, which is especially pronounced with mitochondrial enzymes. Mitochondria are the main producers of ROS, and posttranslational modifications of mitochondrial proteins (especially reversible oxPTMs) are negative feedback loops, which regulate the genesis of ROS at production sites [17]. The A and E subunits of ATP synthase showed reduced levels of S-nitrosylation and S-glutathionylation upon capacitation. Interestingly, five TCA cycle enzymes (aconitase, 2-oxoglutarate dehydrogenase, dihydrolipoyl dehydrogenase, fumarate hydratase and citrate synthase) showed a decrease in the level of S-nitrosylation, and another two TCA enzymes (fumarate hydratase and isocitrate dehydrogenase) showed a simultaneous increase in the level of S-glutathionylation. In somatic cells, reversible S-glutathionylation and S-nitrosylation of mitochondrial proteins serve as functional switches to temporarily inhibit glycolysis, the TCA cycle, oxidative phosphorylation, amino acid metabolism, and fatty acid combustion, resulting in the diversion of fuels for NADPH-producing pathways and the inhibition of ROS production [17, 18]. Generally, the S-nitrosylation and S-glutathionylation of mitochondrial proteins are considered inhibitors of cell metabolism to desensitize hydrogen peroxide signals [17]. Therefore, it can be assumed that metabolic pathways become activated, promoting ROS production during sperm capacitation, since most changes in mitochondrial proteins involve deglutathionylation and denitrosylation. It is also worth noting that among mitochondrial proteins, the ATP synthase C subunit showed reduced levels of tyrosine phosphorylation, and citrate synthase showed increased phosphorylation, which indicates that crosstalk occurs between reversible oxPTMs and phosphorylation, which accompanies sperm capacitation.

Interestingly, previous studies (in which the total changes in reversible oxPTMs due to capacitation were analyzed) showed that proteins related to glycolysis (ENO, ALDOA), the TCA cycle (CS, ME2) and OXPHOS (ATP synthase, cytochrome b-c1) were oxidized, not reduced [10]. The above indicates that the reversible oxidation and reduction of proteins is a complex process and that oxidation of mitochondrial proteins during capacitation applies to reversible oxPTMs other than S-glutathionylation and S-nitrosylation, e.g., the formation of disulfide bridges and sulfoxylation.

### Zona-pellucida binding proteins are potentially redox-regulated during capacitation

**Fig. 12:**
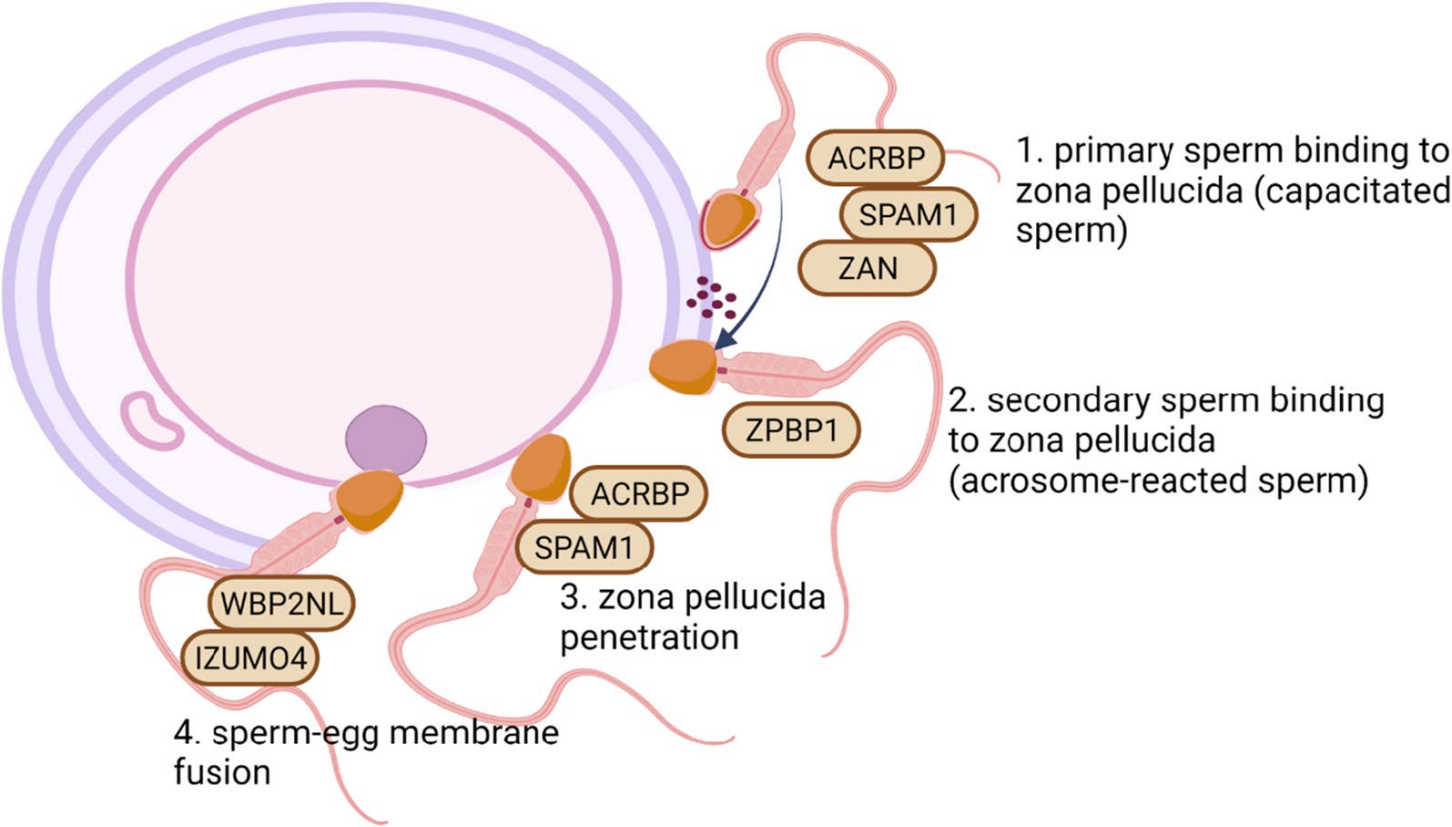
The sequence of sperm–oocyte interaction events. Zona pellucida binding proteins marked with brown ovals underwent S-nitrosylation, S-glutathionylation (and reverse reactions) due to capacitation of the bull sperm

Our results demonstrated for the first time that sperm zona pellucida (ZP)-binding proteins were redox-regulated during capacitation. Zona-pellucida binding proteins that underwent changes in the level of S-glutathionylation and S-nitrosylation were involved in (1) primary ZP binding prior to acrosome disassembly (ACRBP, SPAM1, ZAN) [19–22]; (2) secondary ZP binding after exposing the inner acrosomal membrane (ZPBP1) [23]; (3) zona pellucida penetration through the degradation of hyaluronic acid (SPAM1) [21] and (4) sperm-egg membrane fusion (WBP2NL, IZUMO4) [24, 25] (Fig. 12).

The binding of sperm to the glycoprotein coat is a receptor-mediated event that involves interactions of sperm protein with the complementary ZP glycoproteins, which serve as an interspecies barrier to fertilization [26, 27]. It is worth noting that in our studies, both primary (ACRBP, SPAM1, ZAN) and secondary (ZPBP) ZP-binding proteins showed changes in the levels of S-nitrosylation and S-glutathionylation. Capacitation increased the level of S-nitrosylation of ACRBP as well as S-glutathionylation of SPAM1 and ZPBP and decreased the level of S-nitrosylation of ZAN. Apart from their receptor function, detected ZP binding proteins show activities crucial for sperm-oocyte interactions. ACRBP is an important regulator of proteolytic processing events during packaging, in which acrosomal matrix proteins are disassembled during spermatogenesis and these proteins are released during acrosomal exocytosis [20]. Another ZP-binding protein, SPAM1, is a cell surface hyaluronidase involved in the process by which sperm penetrates the oocyte cumulus matrix. However, the receptor function of this protein may be more important [21], and inhibiting the enzymatic activity only reduces the ability to fertilize [28, 29]. ZAN protein is released from the sperm after the acrosome reaction is induced and probably plays a role in attaching the acrosomal shroud to the zona pellucida [22]. ZPBP1 protein is retained by the inner acrosomal membrane after the acrosome reaction and probably functions exclusively as a secondary ZP binding protein [23].

Our results showed that ZP binding proteins involved in sperm-egg membrane fusion (WBP2NL, IZUMO4) were also redox modified due to capacitation. The S-nitrosylation level of the WBP2NL protein (also known as PAWP) decrease due to capacitation. This unique sperm protein triggers calcium oscillations and pronuclear formation in human and mouse oocytes and is crucial for triggering zygotic development [24]. Moreover, it was shown that WBP2NL promotes meiotic resumption and pronuclear development [30]. The function of IZUMO4, another protein involved in sperm-egg membrane fusion, has not been clearly defined. In our research, IZUMO4 was identified as a redox-regulated protein in 5 proteoforms. Sperm capacitation caused two proteoforms to undergo denitrosylation (spots 47, 48), one proteoform to undergo S-nitrosylation (spot 49) and another two proteoforms (spots 120, 121) of IZUMO4 to undergo deglutathionylation, indicating that the redox regulation of this protein was elaborate. For this reason, unraveling the mechanism of the IZOMO4 role during capacitation would be very challenging. Knowledge about the potential functions of IZUMO 4 is mainly based on the highly homologous IZUMO domain essential for sperm-egg plasma membrane binding and fusion of the IZUMO1 isoform [31]. Interestingly, during the acrosome reaction, the membrane protein IZUMO1 rapidly relocates to the plasma membrane in the equatorial segment of the sperm head, which makes spermatozoon fusion competent [32, 33]. Although little is known about the IZUMO4 isoform, this research and our earlier studies [10] indicated that IZUMO4 is redox-regulated in numerous proteoforms, which suggests that this isoform is activated during bull sperm capacitation.

Our results seem to override the existing knowledge on sperm physiology, as they indicate that the capacitation process not only prepares the sperm for a physiological acrosomal response but also conditions the sperm for directly interacting the oocyte via reversible oxPTMs. Due to the novelty, the physiological functions of capacitation-induced reversible oxPTMs of ZP-binding proteins are unclear. We propose that reversible oxPTMs in the form of S-nitrosylation and S-glutathionylation (and reverse reactions) of ZP-binding proteins may be an important stage that determines the ability of ZP penetration and sperm-oocyte membrane fusion. However, further studies involving in vitro fertilization are necessary to confirm this hypothesis unequivocally.

### Sperm flagellar proteins involved in hyperactive motility

Capacitation was accompanied by an increase in S-nitrosylation and S-glutathionylation of flagellar proteins that comprise outer dense fibers (TEKT2, TEKT3, ODF2), radial spokes (RSPH14), dynein arms (DNALI1) and microtubules (TUBA3A). Moreover, the proteins that build outer dense fibers (ODF2 and TEKT2) were also subjected to tyrosine phosphorylation due to capacitation. Interestingly, the above proteins play an important role in hyperactivation, i.e., the change from linear to curvilinear progressive movement, in which the sperm flagella undergo a large bending amplitude. Hyperactivation is part of the capacitation process; however, its regulatory pathway can operate independently from the pathway that prepares sperm for the acrosome reaction [34]. In the case of bull sperm, CASA analysis is not sufficient to detect hyperactivation in bull sperm due to different signaling pathways regulating sperm capacitation and hyperactivation [35]. Although hyperactivation is not necessary to achieve in vitro fertilization capacity it be critical to the success of in vivo fertilization because it enables the sperm to be released from the wall of the oviduct. It should be emphasized that bull sperm hyperactivation occur independently of capacitation. The signal transduction pathway conducive to capacitation is not sufficient to stimulate hyperactivation. In order to stimulate the hyperactivation of bull sperm movement, a hyperactivation inducer, such as procaine or caffeine, should be added to the capacitating buffer [35].

It is all the more intriguing that flagellar proteins, which are usually associated with hyperactivation of sperm motility, underwent post-translational modifications, although hyperactivation itself has not been registered. Most likely, under the influence of the medium for capacitation, there was an activation of movement—which is characteristic for capacitated bull sperm and, unlike hyperactivation, is characterized by symmetrical beating of the flagellum. The results obtained suggest that altering the level of S-glutathionylation and S-nitrosylation may be important mechanism that prepares sperm to undergo hyperactive motility in mammalian sperm in vivo were additional stimulants of hyperactive motility occures. This assumption is supported by the observation that all identified flagellar proteins that exhibit changes in S-nitrosylation, S-glutathionylation and phosphorylation levels due to capacitation constitute a functional unit for the activity of hyperactive motility. Outer dense fibers (TEKT2, TEKT3, ODF2) are paired with the nine outer microtubule doublets of the axoneme (TUBA3A) and are anchored in a structure called the connecting piece, which is located at the base [36]. ODFs add mechanical stiffness to the flagellum, creating the functional effect of increasing the bending wavelength; for this to occur, more dynein motors (DNALI1) must be entrained. In turn, radial spokes (RSPH14) aid in regulating the activity of dynein motors [37]**.** It is possible that appropriate redox modifications and/or phosphorylation of these proteins provides large torque production, which is crucial to the process of motility hyperactivation.

It seems that ODFs are subject to particularly intense redox modifications during capacitation, which was evidenced in our present and previous studies [10]. Moreover, in the present study, proteins that build outer dense fibers (ODF2 and TEKT2) were also subject to phosphorylation due to capacitation; this result confirms earlier reports in which inhibiting the tyrosine phosphorylation of ODF2 and TEKT2 was associated with impaired motility during the capacitation of hamster spermatozoa [38]. Outer dense fibers detach from the mid piece during capacitation, and failure to detach leads to a stiff mid piece in the sperm tail and poor motility, possibly due to disturbances in the motor response to calcium ions [36]. It is possible that the changes in the S-glutathionylation, S-nitrosylation and phosphorylation levels that were detected are necessary in the process of detaching ODFs. Moreover, ODFs play an active role in sperm hyperactive motility during capacitation, as evidenced by the observation that one isoform of adenylate kinases was localized to ODFs [39]. The obtained results suggest that redox modifications and phosphorylation of proteins included in outer dense fibers, radial spokes, dynein arms and microtubules may be part of the mechanism leading to hyperactive motility. Moreover, ODFs appear to be a particularly important regulatory part of hyperactive motility under capacitive conditions. For this reason, we propose deglutathionylation and tyrosine phosphorylation of ODFs as hallmarks of bull capacitation.

### The regulatory subunit of PKA undergoes tyrosine phosphorylation due to sperm capacitation

Our results showed that three proteoforms of the protein kinase A (PKA) regulatory subunit (spots 347, 348, 349) underwent tyrosine phosphorylation due to sperm capacitation. This modification may be highly important for capacitation, as activation of PKA orchestrates mammalian sperm capacitation, causing downstream PKA substrates to undergo tyrosine phosphorylation, which is a key event by which mammalian sperm acquire the ability to fertilize [40, 41]. The great importance of PKA is evidenced by the observation that proper induction of tyrosine phosphorylation does not occur when PKA is inhibited and there is no compensation for its crucial role [40, 42]. Our results indicated that among various regulatory subunit (PKA-RIα, RIβ, RIIα and RIIβ), PKA-RIα was exclusively phosphorylated during capacitation. In other words, PKA-RIα undergoes tyrosine phosphorylation; however, it remains unclear which mechanism is involved in this phosphorylation. It is possible that PKA-RIIα, and thus PKA activity, could be modulated by an unknown tyrosine kinase or via autophosphorylation [41]. Our results suggest that PKA-RIα, and thus PKA activity, could be modulated by a previously unrecognized molecular mechanism crucial for sperm capacitation. Thus, the present results complement the understanding of PKA regulation, which drives the entire cascade of pTyr events in capacitation and may become the starting point for uncovering the further steps that mediate capacitation signaling.

### Actin-related proteins undergo changes in S-nitrosylation and tyrosine phosphorylation during capacitation

During bull sperm capacitation, polymerization of globular (G)-actin to filamentous (F)-actin occurs, followed by depolymerization in the last phase of capacitation, which precedes the acrosome reaction [43]. The results obtained from cytometric analysis indicated that bull sperm can be in the final stage of capacitation because F-actin levels were reduced due to capacitation (regardless of whether the PRDXs were active or inhibited) (Fig. 2). In the other hand [44], showed a significant increase in F-actin in capacitated bull sperm after 4 h with the decrease in F-actin in acrosome-reacted sperm. It is possible that decrease of F-actin in this study is a consequence of the use of cryopreserved spermatozoa since the actin cytoskeleton is affected by the freezing/thawing process. The change in the F-actin level was accompanied by changes in the S-nitrosylation level of two proteins involved in the actin polymerization process, ACTL7A (denitrosylation) and CAPZB (S-nitrosylation), and an increase in the level of phosphorylation of FSCN3.

CAPZB deserves particular attention since the protein is highly involved in actin polymerization during sperm capacitation [45]. Moreover, two proteoforms of CAPZB were also found to be oxidized during capacitation in a previous study [10], confirming that the oxidative modification of CAPZB is important during capacitation. Perhaps S-nitrosylation of CAPZB may serve to regulate access to the free barbed ends of actin filaments. The specific role of FSCN3 has not been elucidated, but it is possible that the protein enables the binding activity of actin filament (www.genecards.org). Interestingly, a previous study [3] revealed an association between mouse sperm capacitation and an almost threefold increase in the abundance of this protein. Our results suggest that FSCN3 undergoes phosphorylation during bull sperm capacitation, which seems to regulate the actin polymerization/depolymerization process.

In summary, the S-nitrosylation and phosphorylation of a group of actin-related proteins may be part of the mechanism that activates the actin depolymerization process that occurs in the final step of capacitation [43]. Perhaps capacitation-induced posttranslational modifications of CAPZB, ACTL7A and FSCN3 induce functional changes in these proteins, which enables the outer acrosomal membrane and the overlying plasma membrane to move into close proximity and fuse prior to the acrosome reaction.

### Effect of PRDX inhibition on PTMs during capacitation

Our results support that the activity of PRDXs does not affect the intracellular accumulation of calcium ions (Fig. 1b), actin polymerization (Fig. 2) or acrosine availability (Fig. 3). On the other hand, the strong decrease in intracellular NO at the cellular level was most likely related to the significant increase in protein S-nitrosylation observed, which was due to the inhibition of PRDXs (Additional files 4, 5, 6, 7, 8). Moreover, at both the cellular and proteomic levels, the activity of PRDXs is essential for the tyrosine phosphorylation of proteins during capacitation. Previous study demonstrated that inhibition of PRDXs by conoidin A significantly decreased the oxidized form of peroxiredoxins (PRDXs-SO3) in spermatozoa [46]. Decreased PRDX activity was associated with a significant reduction in sperm motility parameters, viability, and intracellular ATP, whereas ROS levels, DNA fragmentation, and loss of mitochondrial membrane potential were increased.

The above results were also confirmed at the proteomic level, since inhibition of PRDXs caused a global increase in S-nitrosylation and S-glutathionylation and a decrease in tyrosine phosphorylation compared to that of sperm with active PRDXs (Fig. 10b). To conclude, our results strongly suggest that there is a relationship between PRDX activity and tyrosine phosphorylation, S-glutathionylation and S-nitrosylation during capacitation. In the following sections, the most significant changes in the formation of PTMs in capacitation, which depend on the activity of PRDXs, will be discussed.

### The inhibition of PRDXs affects the level of S-glutathionylation and S-nitrosylation of redox enzymes during capacitation

Previous studies in which all reversible redox modifications were detected have shown that the level of oxidation within redox enzymes varies greatly [10]. The present results, however, did not show changes in the levels of S-glutathionylation and S-nitrosylation in this group of proteins, which may indicate that redox enzymes undergo reversible oxidative modifications during capacitation other than S-glutathionylation and S-nitrosylation, such as the formation of disulfide bridges or sulfoxylation.

Surprisingly, the inhibition of PRDXs significantly changed the levels of S-glutathionylation and S-nitrosylation of the redox enzymes GAPDHS, GSTO2 and SOD2. The inhibition of PRDXs resulted in an increased level of S-glutathionylation in the three GAPDHS proteoforms compared to that of the non-capacitated and capacitated samples with active PRDXs. Moreover, inhibition of PRDXs resulted in denitrosylation of the enzyme GSTO2 and SOD2, another key enzyme for redox signaling in capacitation. The GAPDHS and GSTO2 enzymes have previously been identified as potential redox signal transducers involved in capacitation [10]. Both enzymes are highly reactive with H_2_O_2_ and possess conserved Cys residues in their active sites. In turn, the SOD enzyme produces H_2_O_2_, which is the most important redox signaling messenger during sperm capacitation. Earlier results showed decreased levels of reversible oxPTMs of SOD2 during capacitation [10]. The present results revealed that S-nitrosylation probably lags behind reversible oxPTM, which exhibits a decreased level during capacitation.

The role of PRDXs as major components in redox signal transmission was recently discovered and documented in somatic cells; it was concluded that PRDXs can protect most proteins against oxidation while actively oxidizing a subset of proteins, depending on site-specific interactions [47–49]. It is speculated that proteins with disordered domains can interact with the center of PRDX rings and are thus brought into the proximity of peroxidatic cysteines. Alternatively, the pairing of thiol peroxidases and target proteins may be facilitated by the supramolecular scaffolding context of larger signaling complexes, potentially involving dedicated adaptor proteins [50]. Indeed, mammalian PRDXs have often been copurified as components of larger signaling complexes [51]. The results of our study indicated for the first time that the activity of PRDXs has a direct impact on the potential redox signal transducers involved in capacitation. The activity of PRDXs may be related to redox signaling, as the activity maintains the appropriate level of GAPDHS S-glutathionylation and the S-nitrosylation of the GSTO2 and SOD2 enzymes.

### Relationship between the redox status of ZP-binding proteins and the activity of PRDXs

We found a significant relationship between the redox status of ZP-binding proteins and the activity of PRDXs, as there are numerous differences between sperm samples in which the PRDX activity was active or inhibited. Such differences were related to the IZUMO4 and ACRBP proteins described above, in which the level of S-nitrosylation was higher when the activity of PRDXs was blocked. Inhibition of PRDXs also caused ADAM2 protein to undergo increased S-nitrosylation, and ADAM2 is a sperm surface protein containing a disintegrin-like domain that binds to integrin receptors on the egg [52]. Based on the above results, it seems that disrupting the activity of PRDX affects the level of reversible oxPTMs in proteins related to sperm-oocyte interactions, which may result in failure of gamete recognition and fertilization.

### Inhibition of PRDXs causes the AKAP proteins to dephosphorylate

PKA can phosphorylate many putative substrates. Therefore, to target the suitable signaling cascade and prevent other substrates from phosphorylating, the regulatory subunit of PKA is tethered by proper scaffolding proteins called AKAPs [53]. Interestingly, inhibition of PRDXs resulted in a decrease in tyrosine phosphorylation of AKAP3 and AKAP4 proteins. This is a very important finding that can explain the decrease in tyrosine phosphorylation of proteins in capacitated, PRDX-inhibited sperm (Fig. 4) (Additional file 9). As mentioned, AKAPs are molecular navigators of PKA anchors due to their physiological substrates [54]. A lack of AKAP3 and AKAP4 results global changes in the sperm proteome, such as mislocalization of PKA and immobility [55]. Both AKAP3 and AKAP4 play very important roles in capacitation; however, they target different signaling complexes. AKAP3 has been found in the acrosomal region, and AKAP4 localizes to the longitudinal columns of the fibrous sheath, restricting the scope of PKA action within a close proximity of motility-related targets in the axoneme [56]. Considering that AKAP3 and AKAP4 play a fundamental role in PKA targeting and downstream tyrosine phosphorylation events in the capacitation process, our results suggest that PRDX activity is necessary to maintain the appropriate level of phosphorylation of AKAP3 and AKAP4 proteins. Presumably, inhibiting PRDX activity inexplicably leads to a decrease in ACAP3 and ACAP4 phosphorylation, disrupting the interaction with PKA and resulting in a decrease in tyrosine phosphorylation during capacitation. Moreover, the same effect may be the reason why sperm motility decreases in PRDX-inhibited capacitated sperm (Table 1), as AKAP4 is likely involved in the signal cascade of phosphorylation in sperm flagella. However, IVF will need to be performed to provide direct evidence.

## Conclusions

Our results revealed that reversible PTMs of sperm proteins occurred during capacitation with the following modifications: S-nitrosylation/denitrosylation, S-glutathionylation/deglutathionylation and tyrosine phosphorylation/dephosphorylation. However, the dominant PTMs detected in capacitated sperm proteins were proteins that underwent denitrosylation, deglutationylation and tyrosine phosphorylation. During capacitation, changes in the level of S-nitrosylation, S-glutathionylation and tyrosine phosphorylation of associated groups of proteins occurred, which indicates that the direction of posttranslational modifications was integrated. Some groups of sperm proteins were associated exclusively with reversible ox PTMs (zona-pellucida binding proteins, redox enzymes and organelle component assembly involved proteins), while others were associated only with phosphorylation (proteins involved in cAMP/PKA pathway) or showed changes in all analyzed types of PTMs (TCA enzymes, energy metabolism enzymes and sperm flagellar), which indicates that PTMs-based regulation of capacitation is a much more complex mechanism than previously assumed.

Sperm mitochondrial proteins were collectively denitrosylated and deglutathionylated, which indicates that metabolic pathways were activated and hydrogen peroxide signals were sensitized during sperm capacitation. Zona-pellucida binding proteins that were particularly rich in reversible ox PTMs were involved in the following sperm–oocyte interaction events: (1) primary ZP binding prior to acrosome disassembly (ACRBP, SPAM1, ZAN), (2) secondary ZP binding after exposing the inner acrosomal membrane (ZPBP1), (3) zona pellucida penetration (SPAM1), and (4) sperm-egg membrane fusion (WBP2NL, IZUMO4). Our results seem to override the existing knowledge on sperm physiology, as they indicate that the capacitation process not only prepares the sperm for a physiological acrosomal response but also conditions the sperm for direct interactions with an oocyte via redox PTMs. We propose that redox PTMs in the form of S-nitrosylation and S-glutathionylation (and reverse reactions) of ZP-binding proteins may be an important stage that determines the ability of ZP penetration and sperm-oocyte membrane fusion.

A large group of sperm flagellar proteins (components of outer dense fibers, radial spokes, dynein arms and microtubules) was associated with all analyzed types of PTMs. This integrated direction of posttranslational modifications was exceptionally significant for ODFs proteins that are involved in capacitation-induced hyperactive motility. For this reason, we propose that deglutathionylation and tyrosine phosphorylation of ODFs are hallmarks of bull sperm capacitation.

For the first time, the obtained results indicate that there is a relationship between PRDX activity and protein phosphorylation, S-glutathionylation and S-nitrosylation, since inhibition of PRDX activity caused a global increase in S-nitrosylation and S-glutathionylation and a decrease in protein phosphorylation compared to that of sperm with active PRDXs. The activity of PRDXs appears to be related to redox signaling during capacitation, as the activity maintains the appropriate levels of S-glutathionylation of GAPDHS and S-nitrosylation of the GSTO2 and SOD2 enzymes. Inhibition of PRDXs also caused dephosphorylation of the AKAP proteins, which may disrupt the interaction between AKAP proteins and PKA, resulting in a decrease in tyrosine phosphorylation during capacitation. Moreover, the same effect may explain the decrease in sperm motility in PRDX-inhibited capacitated sperm. Moreover, a relationship between the redox status of ZP-binding proteins and the activity of PRDXs was shown. Disrupting the PRDX activity affected the level of redox PTMs in proteins related to sperm-oocyte interactions, which may result in failure of gamete recognition and fertilization.

## Supplementary Information

**Additional file 1. Figure S1.** Positive and negative control of 1D S-nitrosylation analysis. 1D gel analysis of relative fluorescence intensity corresponding to the levels of protein S-nitrosylation of bull sperm with negative and positive controls. Lane 1—blocking control, sample without substrate specific reduction with ascorbate. Lane 2—experimental sample, with ascorbate reduction step. Lane 3—positive control, sample treated with NONOate, a nitric oxide donor.

**Additional file 2. Figure S2.** Positive and negative control of 1D S-glutathionylation analysis. 1D gel analysis of relative fluorescence intensity corresponding to the levels of protein S-glutathionylation of bull sperm with negative and positive controls. Lane 1—blocking control, sample without substrate specific reduction with GRX1. Lane 2—experimental sample, with GRX1 reduction step. Lane 3—positive control, sample treated with GSSG, an oxidized glutathione donor.

**Additional file 3. Table S1.** Proteomic data on identified bull sperm proteins.

**Additional file 4. Figure S3.** Representative flow cytometry graphs showing mean fluorescence of Fluo 3-AM corresponding to the level of intracellular calcium in non-capacitated sperm (Non-Cap) (A).

**Additional file 5. Figure S4.** Representative flow cytometry graphs showing flourescence of FITC-conjugated anti-acrosine antibodies corresponding to the level of available acrosine in non-capacitated sperm (Non-Cap) (**A**), capacitated sperm (Cap PRDX+) (**B**) and capacitated sperm with PRDX inhibition (Cap PRDX−) (**C**).

**Additional file 6. Figure S5.** Representative flow cytometry graphs showing nitric oxide-positive and negative sperm populations in non-capacitated sperm (Non-Cap) (**A**), capacitated sperm (Cap PRDX+) (**B**) and capacitated sperm with PRDX inhibition (Cap PRDX−) (**C**).

**Additional file 7. Figure S6.** Representative flow cytometry graphs showing S-glutathionylation-positive and negative sperm populations in non-capacitated sperm (Non-Cap) (**A**), capacitated sperm (Cap PRDX+) (**B**) and capacitated sperm with PRDX inhibition (Cap PRDX−) (**C**).

**Additional file 8. Figure S7.** Representative flow cytometry graphs showing mean fluorescence of FTIC-phalloidin corresponding to the level of actin polymerization in non-capacitated sperm (Non-Cap) (**A**), capacitated sperm (Cap PRDX+) (**B**) and capacitated sperm with PRDX inhibition (Cap PRDX−) (**C**).

**Additional file 9. Figure S8.** Representative histograms showing sperm flagella fluorescence intensity corresponding to the level of tyrosine phosphorylation in non-capacitated sperm (Non-Cap) (**A**), capacitated sperm (Cap PRDX+) (**B**) and capacitated sperm with PRDX inhibition (Cap PRDX-) (**C**).

## Data Availability

All data generated or analysed during this study are included in this published article.

## Abbreviations

ACO2: Aconitase [EC 4.2.1.3]
ADAM2: Disintegrin and metalloproteinase domain-containing protein 2/Beta-Fertilin [EC 3.4.24]
AK: Adenylate kinases [EC 2.7.4.3]
AKAP: A-kinase anchoring proteins [EC 2.7.11.11]
ALDOA: Aldolase A [EC 4.1.2.13]
ATP2: ATP synthase [EC 7.1.2.2]
CK1: Casein kinase [EC 2.7.11.1]
CS: Citrate synthase [EC 2.3.3.1]
DLD: Dihydrolipoyl dehydrogenase [EC 1.8.1.4]
ENO: Enolase [EC 4.2.1.11]
FH: Fumarate hydratase [EC 4.2.1.2]
GAPDHS: Glyceraldehyde-3-phosphate dehydrogenase, spermatogenic [EC 1.2.1.12]
Grx1: Glutaredoxin 1 [EC 1.11.1.15]
GSSG: Glutathione reductase [EC 1.8.1.7]
GSTO2: Glutathione S-transferase omega-2 [EC 2.5.1.18]
IDH: Isocitrate dehydrogenase [EC 1.1.1.42]
ME2: NAD-dependent malic enzyme, mitochondrial [EC 1.1.1.38]
NADPH: Nicotinamide adenine dinucleotide phosphate [EC 1.4.1.13]
OGDH: 2-Oxoglutarate dehydrogenase [EC 1.2.4.2]
PKA: Protein kinase A [EC 2.7.11.11]
PPP: Serine/threonine-protein phosphatase [EC 3.1.3.16]
SOD2: Superoxide dismutase 2, mitochondrial [EC 1.15.1.1]
SPAM1: Hyaluronidase PH-20 [EC 3.2.1.35]
UQCR1: Cytochrome b-c1 [EC 7.1.1.8
