# Supplementary material for: New insights into posttranslational modifications of proteins during bull sperm capacitation
Source: Cell Commun Signal. 2023 Apr 12;21:72. doi: 10.1186/s12964-023-01080-w (PMC10091539; doi:10.1186/s12964-023-01080-w)
Supplement: Supplementary file 2 — Additional file 1. Figure S1. Positive and negative control of 1D S-nitrosylation analysis. 1D gel analysis of relative fluorescence intensity corresponding to the levels of protein S-nitrosylation of bull sperm with negative and positive controls. Lane 1—blocking control, sample without substrate specific reduction with ascorbate. Lane 2—experimental sample, with ascorbate reduction step. Lane 3—positive control, sample treated with NONOate, a nitric oxide donor. [file 12964_2023_1080_MOESM2_ESM.pdf]

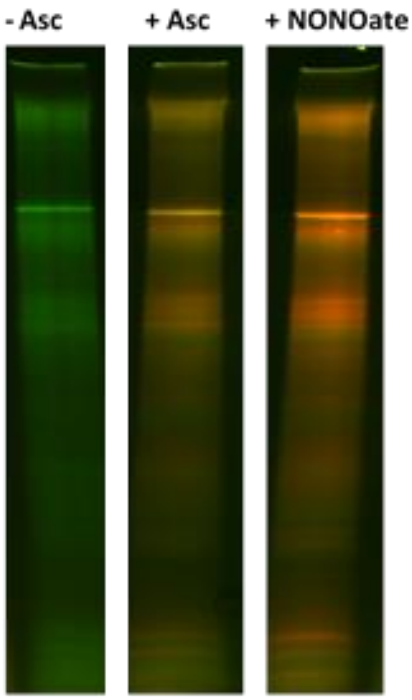

Figure S1. Positive and negative control of 1D S-nitrosylation analysis. 1D gel analysis of relative fluorescence intensity corresponding to the levels of protein S-nitrosylation of bull sperm with negative and positive controls. Lane 1 – blocking control, sample without substrate specific reduction with ascorbate. Lane 2 – experimental sample, with ascorbate reduction step. Lane 3 – positive control, sample treated with NONOate, a nitric oxide donor.
