# Supplementary material for: New insights into posttranslational modifications of proteins during bull sperm capacitation
Source: Cell Commun Signal. 2023 Apr 12;21:72. doi: 10.1186/s12964-023-01080-w (PMC10091539; doi:10.1186/s12964-023-01080-w)
Supplement: Supplementary file 3 — Additional file 2. Figure S2. Positive and negative control of 1D S-glutathionylation analysis. 1D gel analysis of relative fluorescence intensity corresponding to the levels of protein S-glutathionylation of bull sperm with negative and positive controls. Lane 1—blocking control, sample without substrate specific reduction with GRX1. Lane 2—experimental sample, with GRX1 reduction step. Lane 3—positive control, sample treated with GSSG, an oxidized glutathione donor. [file 12964_2023_1080_MOESM3_ESM.pdf]

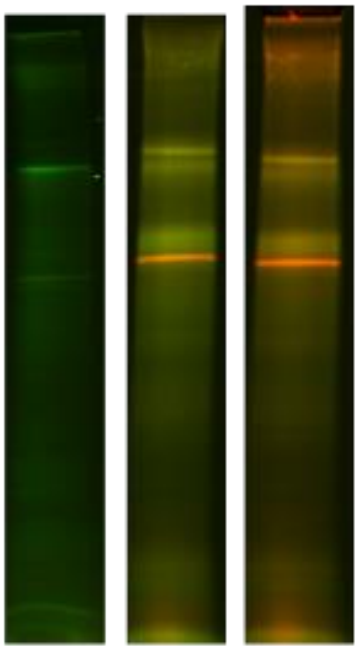

Figure S2. Positive and negative control of 1D S-glutathionylation analysis. 1D gel analysis of relative fluorescence intensity corresponding to the levels of protein S-glutathionylation of bull sperm with negative and positive controls. Lane 1 – blocking control, sample without substrate specific reduction with GRX1. Lane 2 – experimental sample, with GRX1 reduction step. Lane 3 – positive control, sample treated with GSSG, an oxidized glutathione donor.
