# Supplementary material for: New insights into posttranslational modifications of proteins during bull sperm capacitation
Source: Cell Commun Signal. 2023 Apr 12;21:72. doi: 10.1186/s12964-023-01080-w (PMC10091539; doi:10.1186/s12964-023-01080-w)
Supplement: Supplementary file 4 — Additional file 3. Table S1. Proteomic data on identified bull sperm proteins. [file 12964_2023_1080_MOESM4_ESM.pdf]

Tab S1. Proteomic data on identified bull sperm proteins

Spot no	Protein name (gene name)	Accession number	Calculated MW/pI	Protein score/ number of peptides
1	aconitate hydratase, mitochondrial precursor (ACO2)	NP_776402.1	86045/8.08	861/10
2	aconitase 2, mitochondrial precursor (ACO2)	DAA29059.1	86047/7.87	874/11
3	aconitase 2, mitochondrial precursor (ACO2)	DAA29059.1	86047/7.87	566/8
4	2-oxoglutarate dehydrogenase, mitochondrial precursor (OGDH)	NP_001069498.1	116875/6.28	159/2
5	ATP synthase subunit alpha, mitochondrial isoform X1 (ATP5F1A)	XP_024839950.1	59767/9.21	473/4
6	J Chain J, Atp Synthase Subunit Alpha Heart Isoform (ATP5F1A)	2JJ2	55344/7.87	1000/8
7	pyruvate kinase PKM isoform X1 (PKM)	XP_005211367.1	58536/7.62	251/4
8	C Chain C, BOVINE MITOCHONDRIAL F1-ATPASE	1COW	55314/7.87	525/4
9	tektin 3	DAA18702.1	57063/6.69	486/7
10	dihydrolipoyl dehydrogenase, mitochondrial (DLD)	NP_001193099.1	54723/7.59	347/3
11	dihydrolipoyl dehydrogenase, mitochondria (DLD)	NP_001193099.1	54723/7.59	442/5
12	glycerol-3-phosphate dehydrogenase, mitochondrial isoform X1 (GPD2)	XP_024855441.1	81225/6.60	140/2
13	actin-like protein 7A (ACTL7A)	NP_001033659.1	49669/6.29	400/3
14	MICOS complex subunit MIC60 isoform X4 (IMMT)	XP_005212827.1	84930/6.18	186/2
15	T-complex protein 1 subunit alpha isoform X1	XP_024852291.1	58367/5.90	237/2
16	N Chain N, Cytochrome b-c1 complex subunit 1, mitochondrial (UQCRC1)	5NMI	49617/5.47	200/2
17	disintegrin and metalloproteinase domain-containing protein 2 isoform X3 (ADAM2)	XP_024841958.1	73820/5.54	244/2
18	glyceraldehyde-3-phosphate dehydrogenase, testis-specific isoform X1 (GAPDHS)	XP_005219028.1	44475/8.51	344/3

19	B Chain B, Cytochrome B-c1 Complex Subunit 2, Mitochondrial (UQCRC2)	5KLV	46609/7.79	721/6
20	glyceraldehyde-3-phosphate dehydrogenase, testis-specific isoform X1 (GAPDHS)	XP_005219028.1	44475/8.51	307/2
21	N Chain N, Cytochrome b-c1 complex subunit 1, mitochondrial (UQCRC1)	5NMI	49617/5.47	198/2
22	fumarate hydratase, mitochondrial (FH)	DAA21383.1	54827/9.06	490/5
23	fumarate hydratase, mitochondrial (FH)	DAA21383.1	54827/9.06	377/4
24	fumarate hydratase, mitochondrial (FH)	DAA21383.1	54827/9.06	124/2
25	Zonadhesin (ZAN)	XP_024840914.1	306248/6.93	333/4
26	Zonadhesin (ZAN)	XP_024840914.1	306248/6.93	102/2
27	citrate synthase, mitochondrial precursor (CS)	DAA29729.1	51968/8.16	164/2
28	citrate synthase, mitochondrial precursor (CS)	DAA29729.1	51968/8.16	174/2
29	uncharacterized protein C6orf163 homolog (C6orf163)	NP_001073240.1	39253/6.59	144/2
30	dnaJ homolog subfamily B member 13 (DNAJB13)	NP_001029708.1	36226/7.66	394/4
31	F-actin-capping protein subunit beta isoform X3 (CAPZB)	XP_024855040.1	30952/5.69	331/4
32	postacrosomal sheath WW domain-binding protein (WBP2NL)	NP_001075910.1	32231/5.61	369/5
33	radial spoke head 14 homolog isoform X1	XP_024833234.1	37624/5.81	121/2
35	serine/threonine-protein phosphatase PP1-gamma catalytic subunit isoform X1 (PPP1CC)	XP_005217794.1	39265/5.80	479/5
36	B Chain B, Serum albumin (ALB)	6RJV	68416/5.60	219/3
37	serine/threonine-protein phosphatase PP1-gamma catalytic subunit isoform X1 (PPP1CC)	XP_005217794.1	39265/5.80	245/3
38	acrosin-binding protein isoform X1 (ACRBP)	XP_002687915.1	62374/4.99	302/3
39	NADH dehydrogenase [ubiquinone] iron-sulfur protein 8, mitochondrial isoform X3 (NDUFS8)	XP_005227041.1	24394/6.45	301/3
40	ubiquitin carboxyl-terminal hydrolase isozyme L1 isoform X1 (UCHL1)	XP_005207929.1	25265/5.07	85/2

41	glutathione S-transferase omega-2 isoform X1 (GSTO2)	XP_024841561.1	28869/7.49	323/4
42	superoxide dismutase [Mn], mitochondrial precursor (Sod2)	NP_963285.2	24794/8.70	327/3
43	izumo sperm-egg fusion protein 4 (IZUMO4)	NP_001095398.1	18261/5.94	446/5
44	izumo sperm-egg fusion protein 4 (IZUMO4)	NP_001095398.1	18261/5.94	356/3
45	superoxide dismutase [Mn], mitochondrial precursor (Sod2)	NP_963285.2	24794/8.70	263/2
46	acrosin-binding protein isoform X1 (ACRBP)	XP_002687915.1	62374/4.99	157/2
47	izumo sperm-egg fusion protein 4 (IZUMO4)	NP_001095398.1	18261/5.94	406/5
48	izumo sperm-egg fusion protein 4 (IZUMO4)	NP_001095398.1	18261/5.94	375/5
49	izumo sperm-egg fusion protein 4 (IZUMO4)	NP_001095398.1	18261/5.94	204/3
50	superoxide dismutase [Mn], mitochondrial precursor (Sod2)	NP_963285.2	24794/8.70	110/2
51	ras-related protein Rab-2A (RAB2A)	NP_001068822.1	21009/7.01	371/4
52	nucleoside diphosphate kinase homolog 5 isoform X1 (NME5)	XP_005209449.1	23907/6.31	450/5

Spot no	Protein name (gene name)	Accession number	Calculated MW/pI	Protein score/ number of peptides
100	sperm adhesion molecule 1 isoform X2 (SPAM1)	XP_024846271.1	60267/8.90	570/6
101	sperm adhesion molecule 1 isoform X2 (SPAM1)	XP_024846271.1	60267/8.90	488/7
102	glyceraldehyde-3-phosphate dehydrogenase, testis-specific isoform X1 (GAPDHS)	XP_005219028.1	44475/8.51	265/3
103	glyceraldehyde-3-phosphate dehydrogenase, testis-specific (GAPDHS)	NP_001035642.1	43659/8.32	160/2
104	B Chain B, Cytochrome B-c1 Complex Subunit 2, Mitochondrial (UQCRC2)	5KLV	46609/7.79	232/3
105	casein kinase II subunit alpha (CSNK2A1)	DAA23123.1	42524/7.29	150/2
106	zona pellucida-binding protein 1 isoform X1 (Zpbp)	XP_005205168.1	40313/9.14	352/4
107	glyceraldehyde-3-phosphate dehydrogenase (GAPDH)	NP_001029206.1	36073/8.50	128/2
108	dynein, axonemal, light intermediate chain 1-like (DNALI1)	DAA31163.1	33525/8.61	263/3
109	isoaspartyl peptidase/L-asparaginase (ASRGL1)	NP_001070503.1	32372/7.00	160/2
110	Phosphatidylethanolamine-Binding Protein	1B7A	20956/7.38	657/5
111	fumarate hydratase, mitochondrial (FH)	DAA21383.1	54827/9.06	752/8
112	fumarate hydratase, mitochondrial (FH)	DAA21383.1	54827/9.06	345/4
113	fumarate hydratase, mitochondrial (FH)	DAA21383.1	54827/9.06	124/2
114	alpha enolase (ENO1)	AF149256_1	47589/6.44	449/4
116	L-lactate dehydrogenase C chain isoform X2 (LDHC)	XP_024843001.1	29973/7.00	222/2
117	fructose-1,6-bisphosphatase, partial (FBP1)	AAZ99065.1	5092/8.21	216/3
118	isocitrate dehydrogenase [NAD] subunit alpha, mitochondrial precursor (IDH3A)	DAA17504.1	40100/6.51	406/4
119	20 alpha-hydroxysteroid dehydrogenase, partial	AAB25333.1	35384/5.81	157/2
120	izumo sperm-egg fusion protein 4 (Izumo4)	NP_001095398.1	18261/5.94	470/4

121	izumo sperm-egg fusion protein 4 (Izumo4)	NP_001095398.1	18261/5.94	460/5
122	ras-related protein Rab-2A isoform X3 (RAB2A)	XP_005215509.1	23675/6.08	305/4
123	E Chain E, ATP SYNTHASE D CHAIN, MITOCHONDRIAL (ATP5ME)	2CLY	18607/6.02	160/2
124	outer dense fiber protein 2 isoform X20 (ODF2)	XP_024854775.1	73285/7.19	121/2
125	leucine zipper protein 2 (LUZP2)	NP_001095806.1	30898/5.61	118/2
126	prohibitin isoform X1 (PHB)	XP_005220626.1	29843/5.57	143/2
127	F Chain F, Atp Synthase Subunit Beta, Mitochondrial (ATP5F1B)	2XND	50283/4.96	1200/10
128	ropporin-1 (ROPN1)	DAA33434.1	24134/5.33	439/6
129	ropporin-1 (ROPN1)	NP_001069368.1	24125/5.22	96/2
130	transitional endoplasmic reticulum ATPase (VCP)	NP_001029466.1	89958/5.13	102/2
131	alpha-1B-glycoprotein precursor (A1BG)	NP_001039708.1	54091/5.29	589/6
132	tubulin alpha-3 chain isoform X3 (Tuba3a)	XP_024833647.1	46605/4.99	753/6
133	60 kDa heat shock protein, mitochondrial (HSPD1)	NP_001160082.1	61110/5.71	330/3
134	carboxypeptidase Q isoform X3 (CPQ)	XP_015330025.1	51222/5.53	335/5
135	heat shock-related 70 kDa protein 2 (HSPA2)	NP_776769.1	69499/5.32	492/6
136	stress-70 protein, mitochondrial precursor (HSPA9)	NP_001029696.1	73981/5.97	310/4
137	B Chain B, Serum albumin (ALB)	6RJV	68416/5.60	249/2
138	B Chain B, Serum albumin (ALB)	6RJV	68416/5.60	487/5
139	B Chain B, Serum albumin (ALB)	6RJV	68416/5.60	791/8
140	B Chain B, Serum albumin (ALB)	6RJV	68416/5.60	610/6
141	glyceraldehyde-3-phosphate dehydrogenase, testis-specific isoform X1 (GAPDHS)	XP_005219028.1	44475/8.51	356/4
142	A Chain A, Actin, Cytoplasmic 1 (ACTB)	3UB5	41921/5.29	102/2
143	C Chain C, NADH dehydrogenase [ubiquinone] iron-sulfur protein 3, mitochondrial (NDUFS3			
