# Supplementary material for: New insights into posttranslational modifications of proteins during bull sperm capacitation
Source: Cell Commun Signal. 2023 Apr 12;21:72. doi: 10.1186/s12964-023-01080-w (PMC10091539; doi:10.1186/s12964-023-01080-w)
Supplement: Supplementary file 5 — Additional file 4. Figure S3. Representative flow cytometry graphs showing mean fluorescence of Fluo 3-AM corresponding to the level of intracellular calcium in non-capacitated sperm (Non-Cap) (A). [file 12964_2023_1080_MOESM5_ESM.pdf]

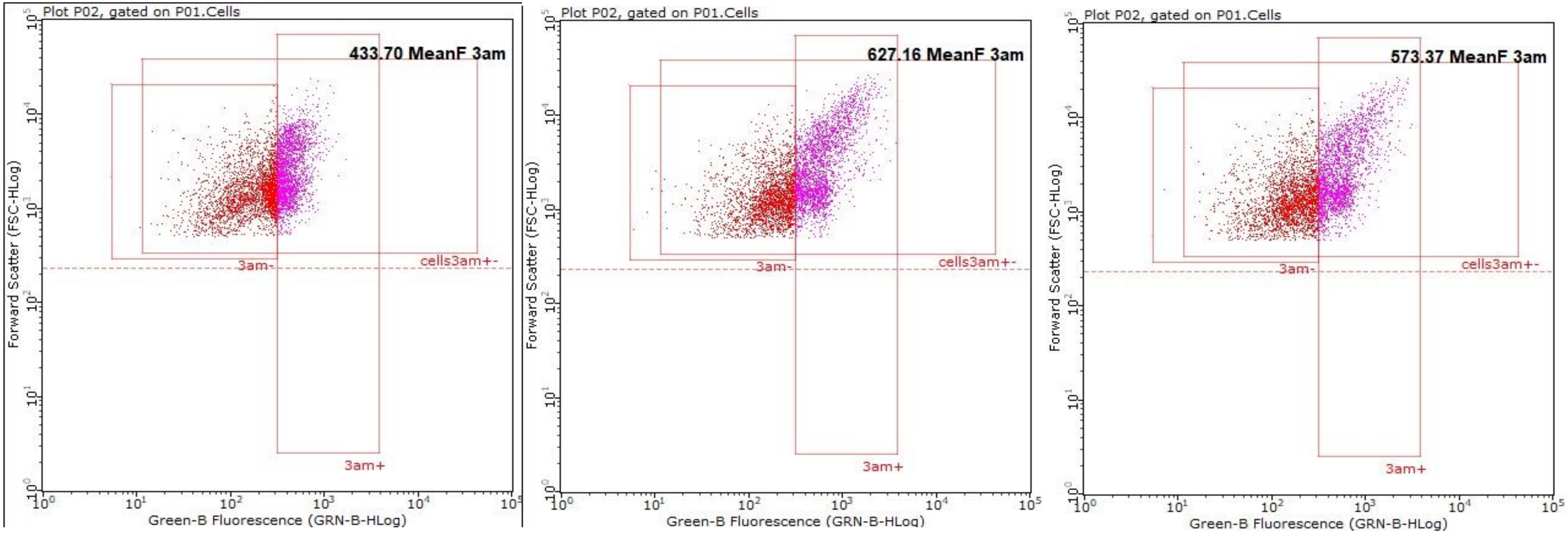

A

B

Fig. S3. Representative flow cytometry graphs showing mean fluorescence of Fluo 3-AM corresponding to the level of intracellular calcium in non-capacitated sperm (Non-Cap) (A),
