# Supplementary material for: New insights into posttranslational modifications of proteins during bull sperm capacitation
Source: Cell Commun Signal. 2023 Apr 12;21:72. doi: 10.1186/s12964-023-01080-w (PMC10091539; doi:10.1186/s12964-023-01080-w)
Supplement: Supplementary file 10 — Additional file 9. Figure S8. Representative histograms showing sperm flagella fluorescence intensity corresponding to the level of tyrosine phosphorylation in non-capacitated sperm (Non-Cap) (A), capacitated sperm (Cap PRDX+) (B) and capacitated sperm with PRDX inhibition (Cap PRDX-) (C). [file 12964_2023_1080_MOESM10_ESM.pdf]

A

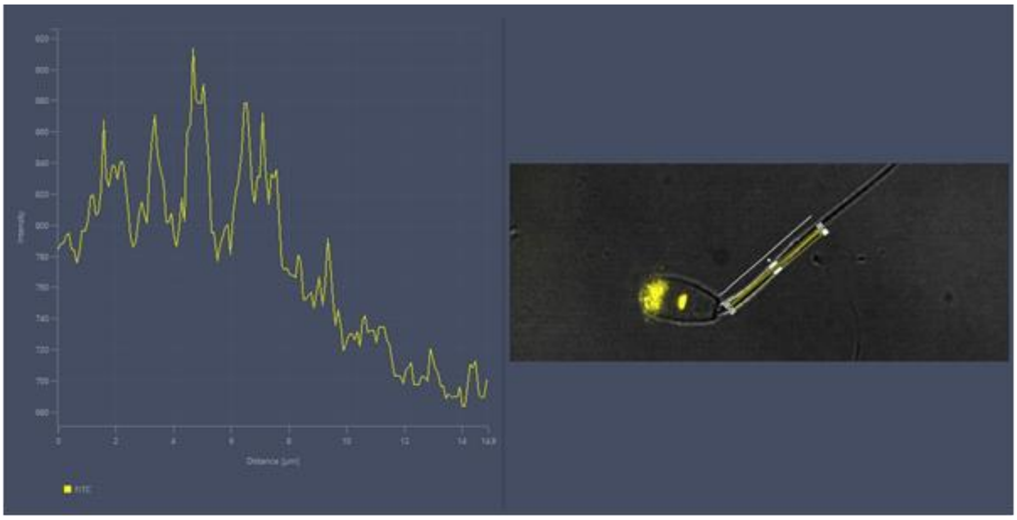

B

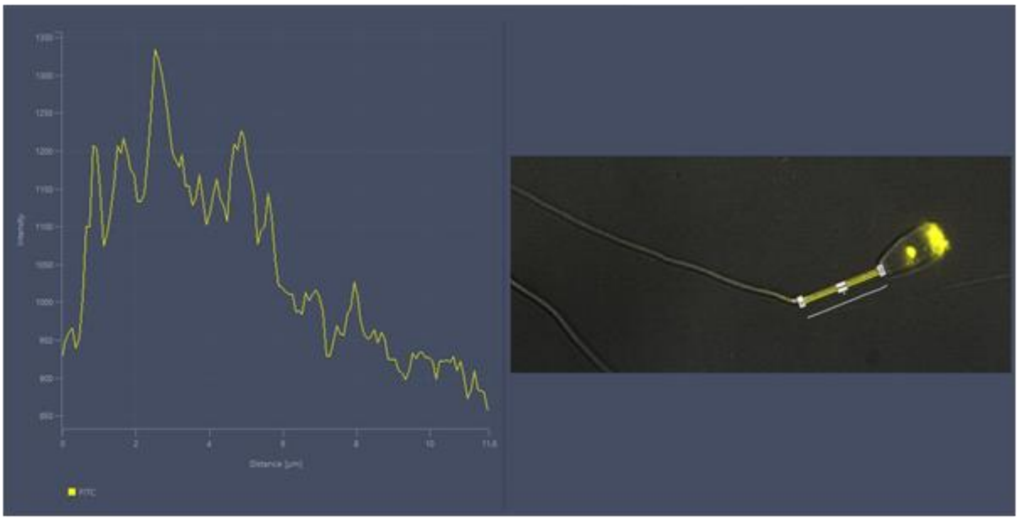

C

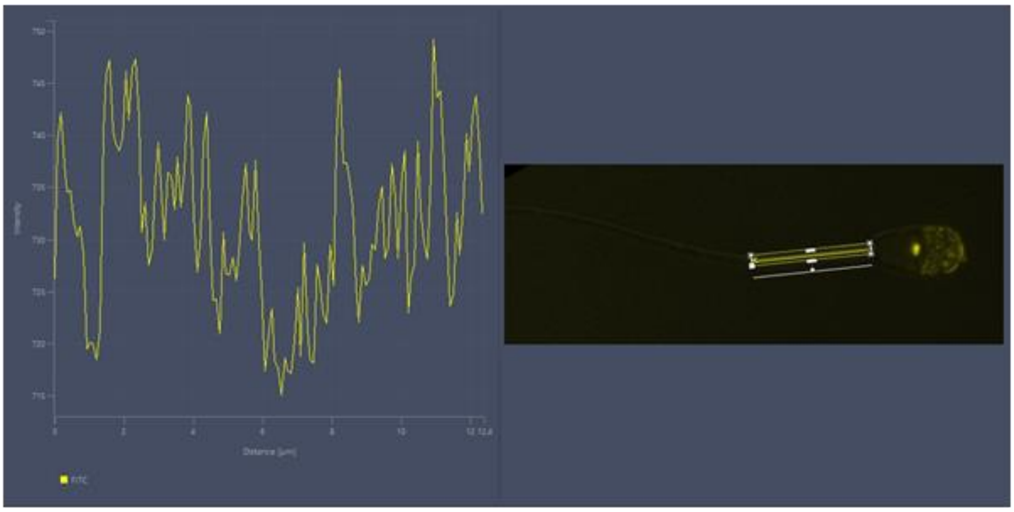

Fig. S8. Representative histograms showing sperm flagella fluorescence intensity corresponding to the level of tyrosine phosphorylation in non-capacitated sperm (Non-Cap) (A), capacitated sperm (Cap PRDX+) (B) and capacitated sperm with PRDX inhibition (Cap PRDX-) (C).
